# A genetic mosaic screen identifies genes modulating Notch signaling in *Drosophila*

**DOI:** 10.1371/journal.pone.0203781

**Published:** 2018-09-20

**Authors:** Luming Ren, Dongqing Mo, Yunlong Li, Tong Liu, Huan Yin, Na Jiang, Junzheng Zhang

**Affiliations:** Department of Entomology and MOA Key Lab of Pest Monitoring and Green Management, College of Plant Protection, China Agricultural University, Beijing, China; National Institutes of Health, UNITED STATES

## Abstract

Notch signaling is conserved in most multicellular organisms and plays critical roles during animal development. The core components and major signal transduction mechanism of Notch signaling have been extensively studied. However, our understanding of how Notch signaling activity is regulated in diverse developmental processes still remains incomplete. Here, we report a genetic mosaic screen in *Drosophila melanogaster* that leads to identification of Notch signali ng modulators during wing development. We discovered a group of genes required for the formation of the fly wing margin, a developmental process that is strictly dependent on the balanced Notch signaling activity. These genes encode transcription factors, protein phosphatases, vacuolar ATPases and factors required for RNA transport, stability, and translation. Our data support the view that Notch signaling is controlled through a wide range of molecular processes. These results also provide foundations for further study by showing that Me31B and Wdr62 function as two novel modulators of Notch signaling activity.

## Introduction

First identified in *Drosophila melanogaster*, the highly conserved Notch signaling pathway is required for cell fate specification in most, if not all, tissues during animal development [1]. Furthermore, Notch signaling also regulates stem cell maintenance and tissue homeostasis in adult life [2]. In keeping with its pleiotropic function in many cell types, dysregulation of Notch signaling in human leads to birth defects as well as tumor formation in various organs [3].

Despite its broad function in many spatially and temporally distinct developmental contexts, the Notch signaling pathway contains only a small number of core components [4]. The *Notch* gene encodes a transmembrane receptor protein which is trans-activated by its ligands, Delta (Dl) and/or Serrate, from neighboring cells. Activation of the Notch receptor triggers a sequence of proteolytic events that releases the Notch intracellular domain (NICD). The NICD subsequently translocates into the nucleus, where it forms an active transcription complex with Suppressor of Hairless [Su(H)] and Mastermind proteins and turns-on the expression of downstream target genes. In the absence of NICD, Su(H) recruits co-repressors to suppress the transcription of Notch target genes [5].

In addition to the components in the core pathway, numerous genes are found to fine-tune the Notch pathway in a context specific manner [4]. These genes encode auxiliary proteins that regulate the amount of Notch receptor and ligands, the proteolytic processing to generate active NICD, the formation of transcriptional active or repressive complexes on the chromatin, as well as trafficking of both the receptor and ligands [4, 5]. Thus, the functional diversity of Notch pathway is generated at different signal transduction steps by the modulatory factors.

The patterning of the adult fly wing blade represents the historical system for studying Notch signaling *in vivo* [6]. Notch signaling is crucial for several major developmental events in the wing, including vein differentiation, wing margin formation and sensory neuronal cell fate determination. We hypothesized that additional regulators may act to support the delicate roles of Notch signaling in these distinct developmental events. Therefore, we performed a genetic mosaic screen to identify genes that modulate Notch signaling activity during fly wing margin formation. We identified nine genes that exert regulatory function at various steps of Notch signal transduction. Other than several well-known components, four of them were poorly studied for regulation of Notch signaling during wing development. More importantly, we discovered two novel modulators of Notch signaling pathway.

## Materials and methods

### Fly genetics

Flies were maintained in standard medium and stocks were kept at room temperature (21–23°C). Crosses were performed at 25°C. Mutant alleles on FRT-containing chromosome were obtained from the Kyoto Drosophila Stock Center (BruinFly collection) [7]. The *Ubx-FLP;Ubi- mRFP*, *FRT40A* stock was used to generate mosaic clones in the developing wing. The *hs-Flp;M(2)24F*, *arm-LacZ*, *FRT40A/Cyo* stock was used to generate large clones in the *Minute* background [8]. Three days old larval were heat-shocked at 37°C for 1 hour to induce mosaic clones. Molecularly defined Deficiency stocks (7818, 9503, 7778, 7779, 8000) and RNAi line against *me31B* (33675) were obtained from the Bloomington Stock Center. Additional Deficiency stocks (150096, 150067) were obtained from the Kyoto Stock Center. RNAi line for *Wdr62* (7337R-I) was provided by the National Institute of Genetics (NIG) in Japan. The *Hh-Gal4*, *UAS-GFP*/*TM6B* and *dpp-Gal4*, *UAS-GFP*/*TM6B* stocks were used to drive transgenic RNAi flies.

### Screen design and phenotypes scoring

Males of each mutant allele from the BruinFly collection were crossed with the *Ubx-FLP;Ubi- mRFP*, *FRT40A* virgins. For the primary screen, at least 100 F1 progenies of each cross were examined for wing developmental defects. Phenotypes with penetration rates higher than 5% were recorded. Mutants that displayed wing margin nicking phenotypes were selected for secondary screen, during which Notch signaling activity was monitored in third-instar larval wing discs. The expression level of Notch signaling target genes Cut and Wingless (Wg) were visualized by immunostaining. Mutants affecting Cut or Wg expression were considered as Notch signaling modulators, and their effects on Notch and Delta were further examined. For mutants that led to disc distortion, clones were generated in the *Minute* background. Wild-type distribution pattern of Cut, Wg, Notch and Delta proteins are shown as control.

### Wing imaginal disc immunostaining and microscopy

**Fig 1:**
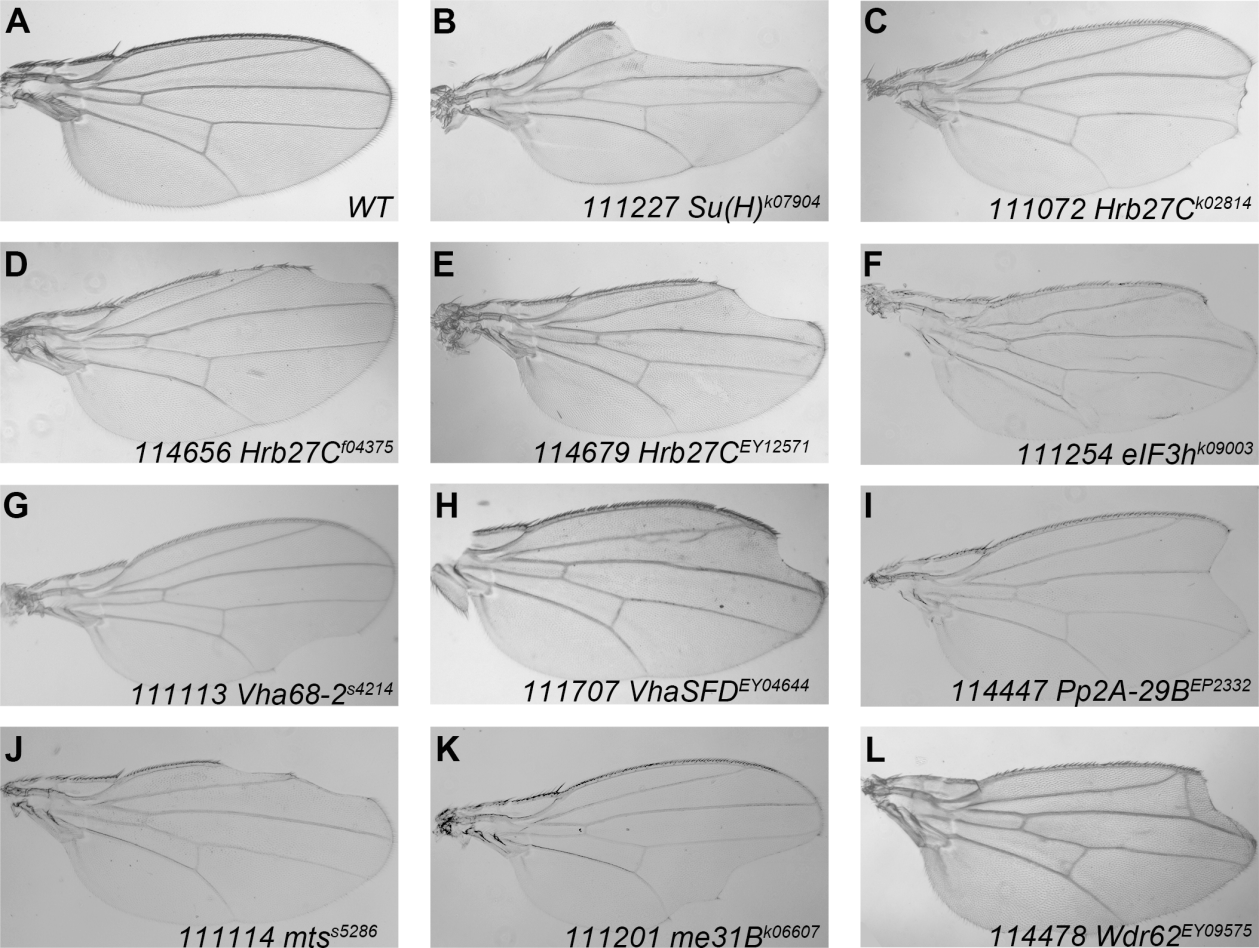
Putative Notch signaling modulators regulate wing margin formation. (A) The adult wing of parental *Ubx-FLP;Ubi- mRFP*, *FRT40A* stock is used as wild type control. Induction of *Su(H)* (B), *Hrb27C* (C, D, E), *eIF3h* (F), *Vha68-2* (G) *VhaSFD* (H), *Pp2A-29B* (I), *mts* (J), *me31B* (K) and *Wdr62* (L) mutant clones in the wing blade led to defective wing margin formation, with different degrees of severity. Specific BruinFly allele and corresponding DGRC stock number are labeled for each mutation.

**Fig 2:**
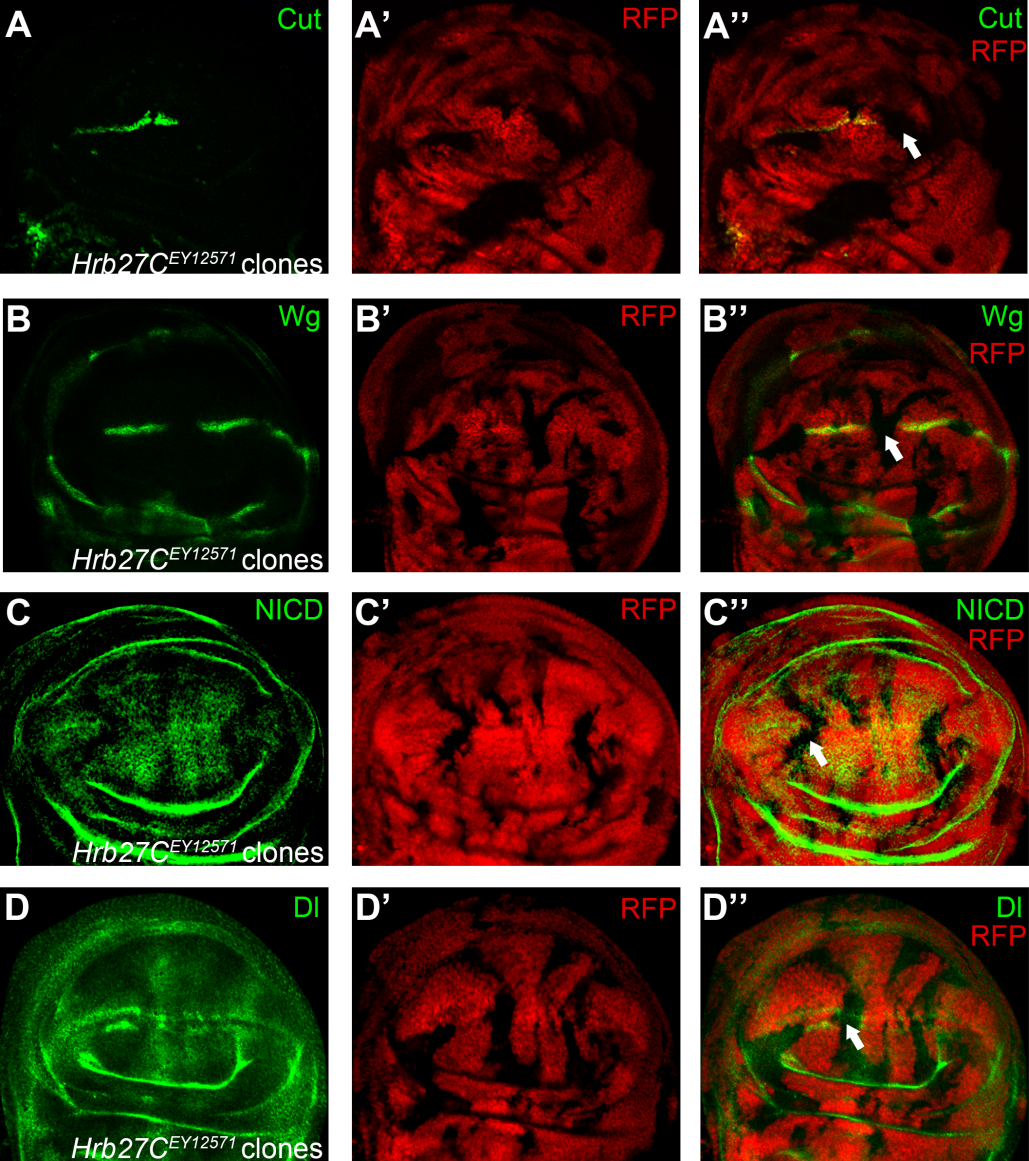
Reduced expression of Hrb27C leads to defective Notch signaling. Expression of Notch signaling targets Cut (A) and Wg (B) are abolished in *Hrb27C*^*EY12571*^ homozygous mutant clones (marked by absence of RFP). (C) Notch protein level is attenuated in *Hrb27C*^*EY12571*^ clones. (D) Reduction of Dl expression is evident in *Hrb27C*^*EY12571*^ mutant cells located at the DV boundary. Representative mutant clones are indicated by arrows.

**Fig 3:**
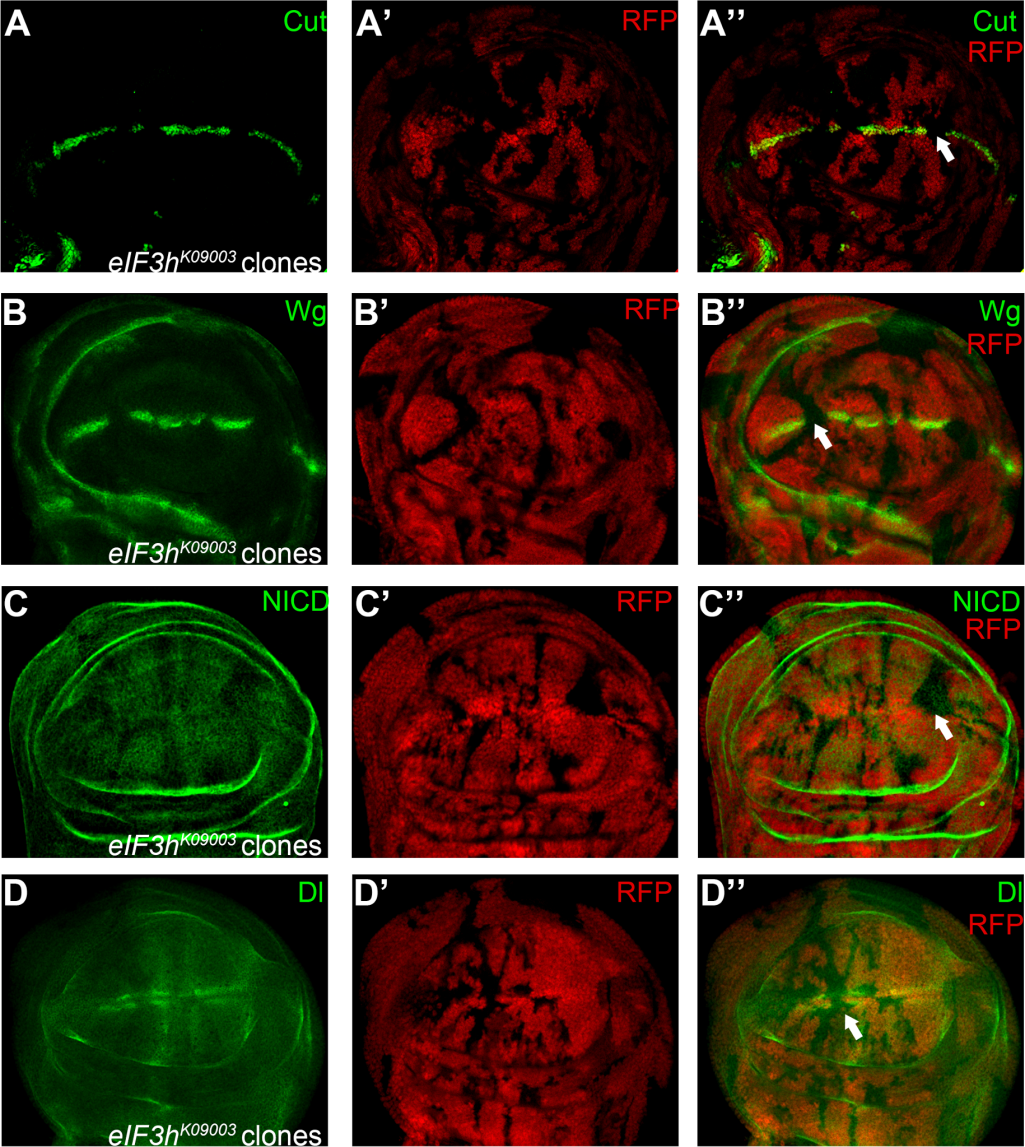
eIF3h positively regulates Notch signaling in the wing disc. Expression of Notch signaling targets Cut (A) and Wg (B) are eliminated in *eIF3h*^*K09003*^ homozygous mutant clones (marked by absence of RFP). Notch protein level (C) is substantially reduced in *eIF3h*^*K09003*^ clones. Dl expression (D) is reduced in clones of *eIF3h*^*K09003*^ mutant cells located at the DV boundary. Representative mutant clones are indicated by arrows.

**Fig 4:**
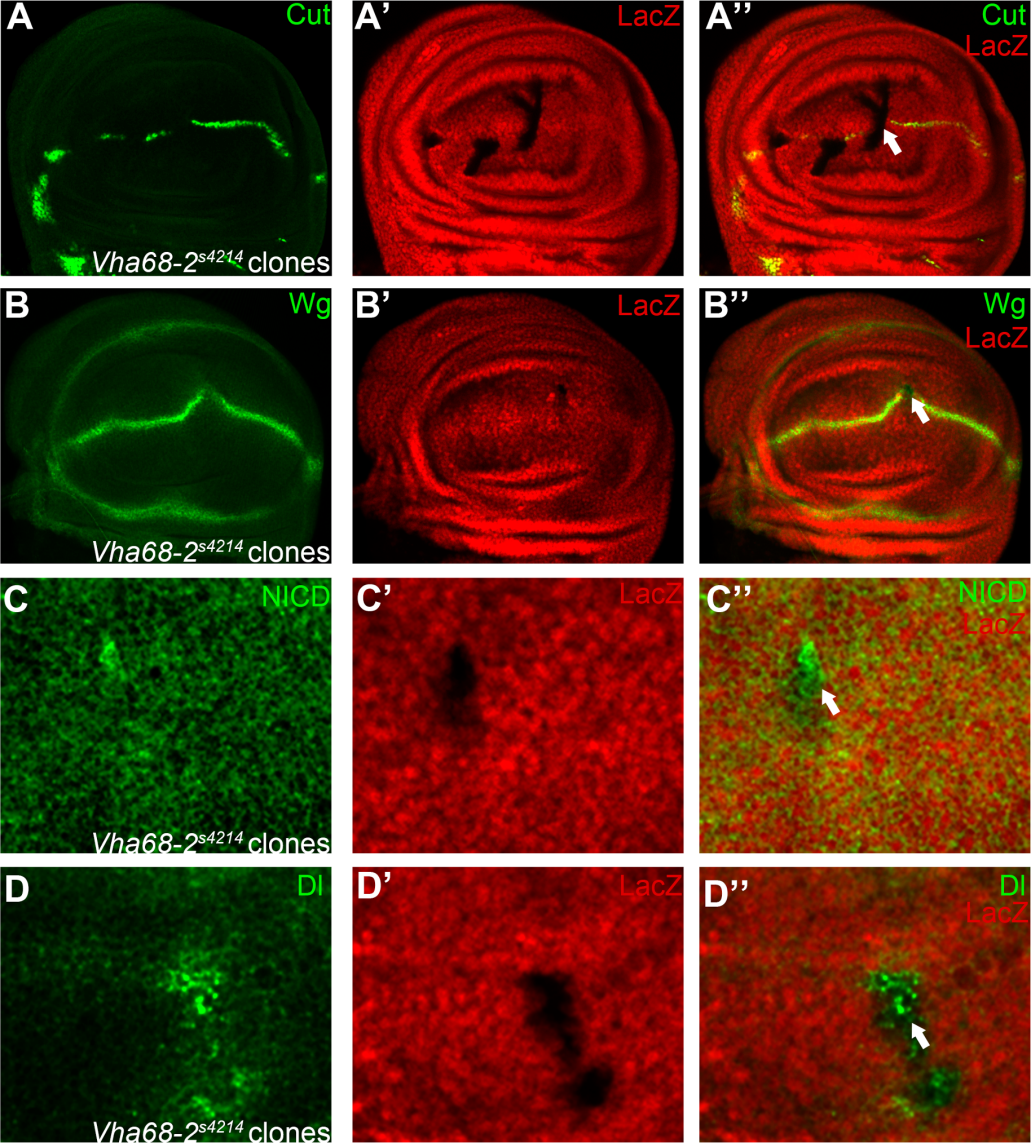
V-ATPase subunit Vha68-2 is a modulator of Notch signaling in the wing. Expression of Notch signaling targets Cut (A) and Wg (B) are reduced in *Vha68-2* ^*s4214*^ homozygous mutant clones (marked by absence of LacZ staining). Both Notch (C) and Dl (D) proteins are accumulated in a subset of *Vha68-2* ^*s4214*^ mutant cells. Representative mutant clones are indicated by arrows. Panels C and D are higher magnification of a small portion of S4 Fig. Clones presented here are generated in the *Minute* background. Phenotype shown in panels A (7 of 8 discs) and B (8 of 11 discs) are consistent, while accumulation of Notch and Dl shows variation among clones.

**Fig 5:**
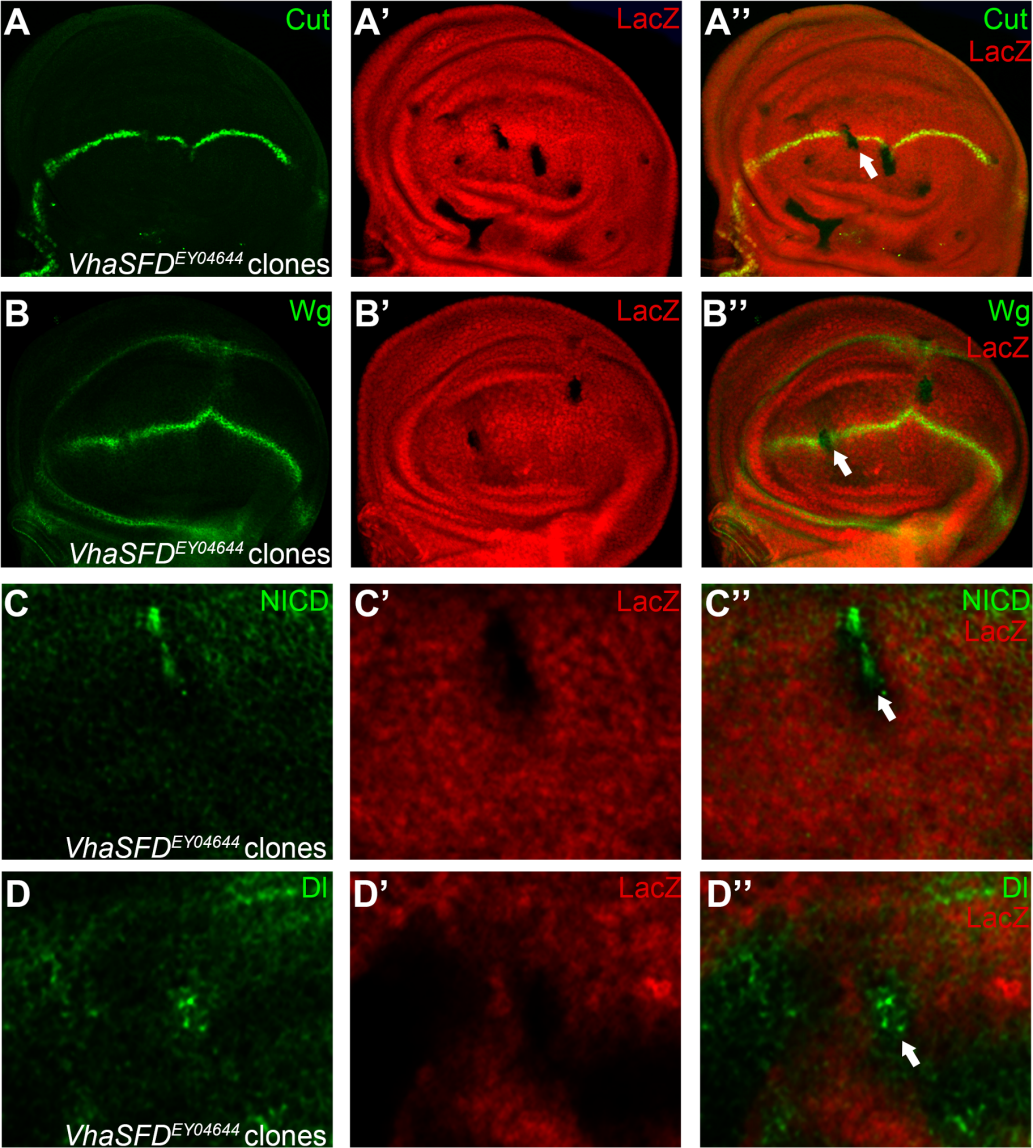
VhaSFD is involved in Notch signaling regulation. Expression of Cut (A) and Wg (B) are reduced in *VhaSFD*^*EY04644*^ homozygous mutant clones (marked by absence of LacZ staining). Notch (C) and Dl (D) proteins are accumulated in a subset of *VhaSFD*^*EY04644*^ mutant cells. Representative mutant clones are indicated by arrows. Panels C and D are higher magnification of a small portion of S4 Fig. Clones presented here are generated in the *Minute* background. Phenotype shown in panels A (9 of 12 discs) and B (6 of 9 discs) are consistent, while accumulation of Notch and Dl shows variation among clones.

**Fig 6:**
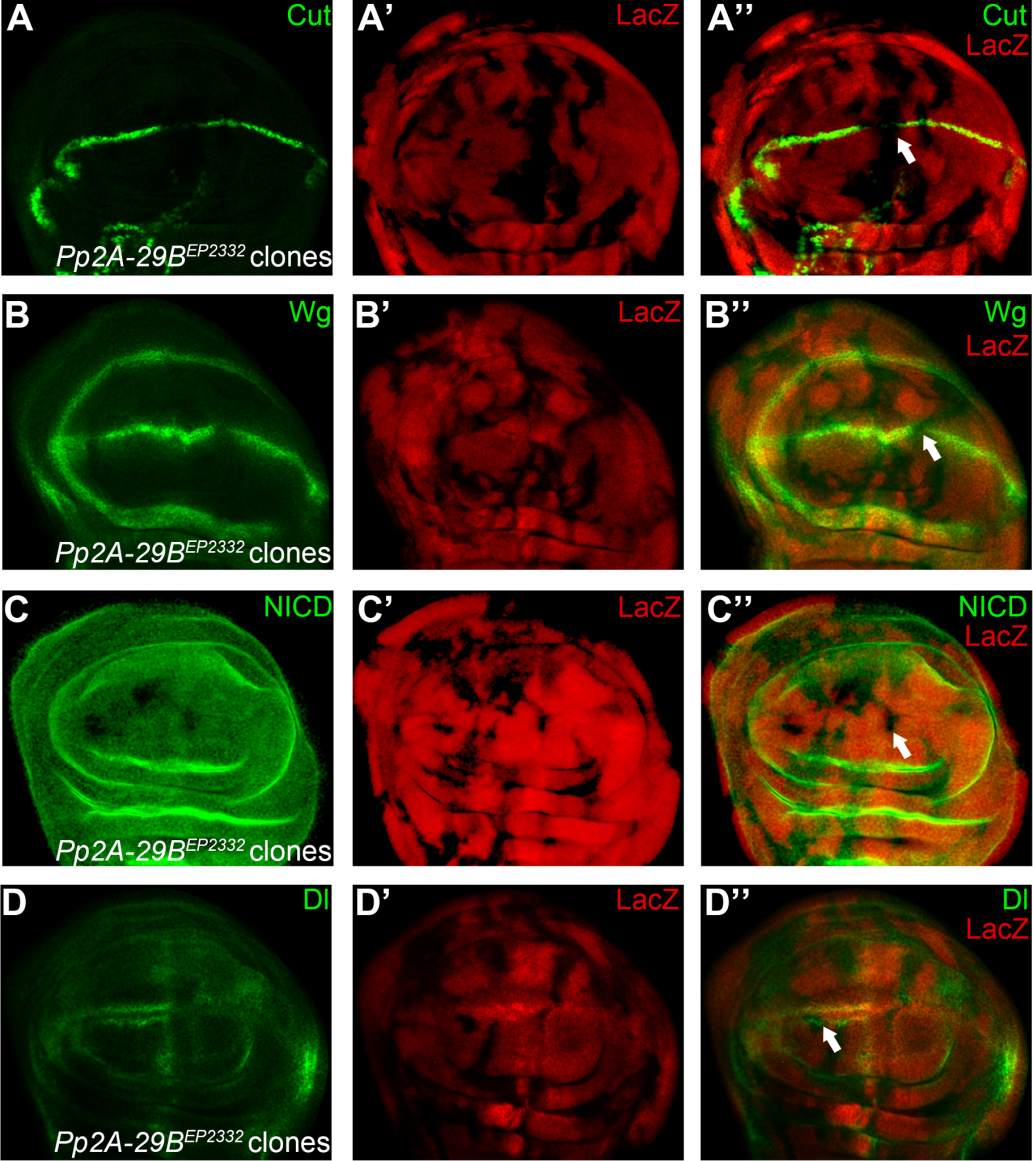
PP2A activity is essential for proper Notch signaling output. Cells in the *Pp2A-29B*^*EP2332*^ homozygous mutant clones lack expression of Cut (A) and Wg (B) at the DV boundary (marked by absence of LacZ staining). Reduced expression of Notch (C) proteins are observed in a subset of *Pp2A-29B*^*EP2332*^ mutant cells. Dl proteins are largely unaffected in *Pp2A-29B*^*EP2332*^ mutant clones (D). Clones presented here are generated in the *Minute* background. Representative mutant clones are indicated by arrows.

**Fig 7:**
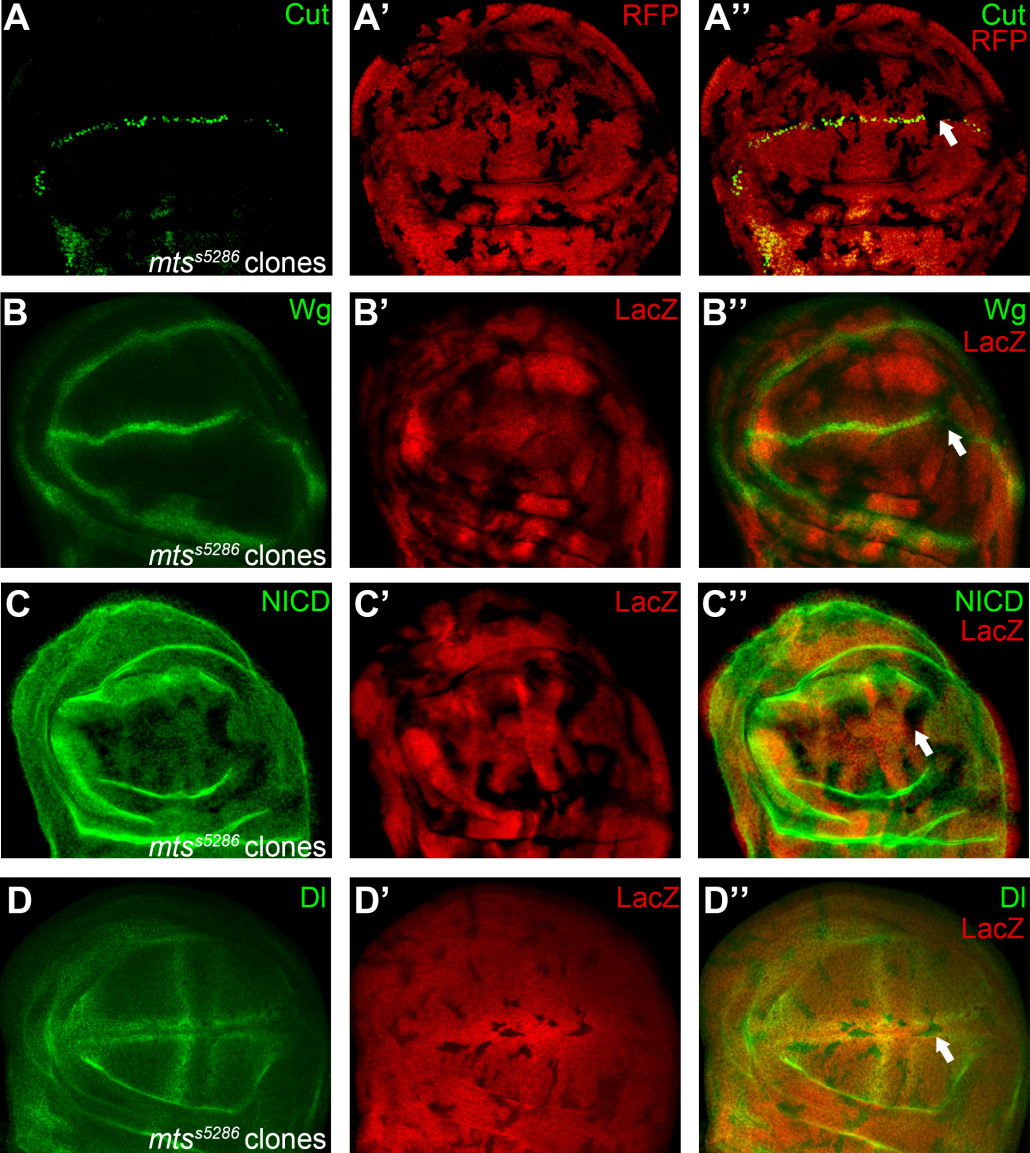
Mts regulates Notch signaling in the wing imaginal disc. Expression of Notch signaling target Cut (A) and Wg (B) are significantly decreased in *mts*^*s5286*^ homozygous mutant clones (marked by absence of RFP or LacZ staining). Reduction of Notch (C) proteins are found in a subset of *mts*^*s5286*^ mutant cells, while Dl (D) proteins pattern remains unchanged. Clones presented here are generated in the *Minute* background except for panel A. Representative mutant clones are indicated by arrows.

**Fig 8:**
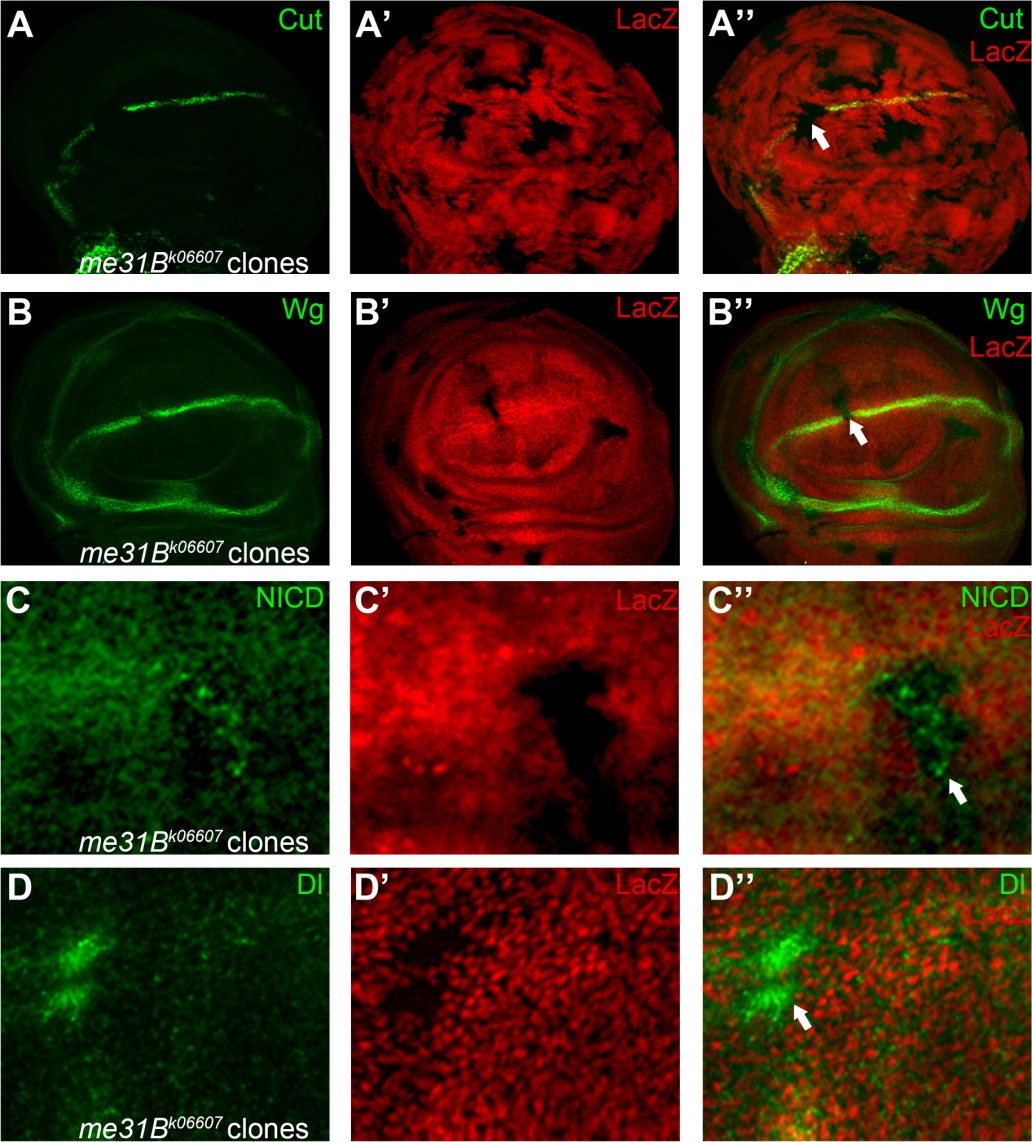
Me31B is a novel regulator of Notch signaling. Expression of Notch signaling targets Wg (A) and Cut (B) are abolished in *me31B*^*k06607*^ homozygous mutant clones (marked by absence of LacZ staining). In *me31B*^*k06607*^ mutant cells, Notch (C) and Dl (D) proteins are accumulated as cellular puncta. Panels C and D are higher magnification of a small portion of S5 Fig. Clones presented here are generated in the *Minute* background. Note that reduction of Cut and Wg are fully penetrating (N>10). Formation of Notch and Dl puncta are consistent among discs (N>15), but the degree of accumulation varies for cells inside the same clone. Representative mutant clones are indicated by arrows.

**Fig 9:**
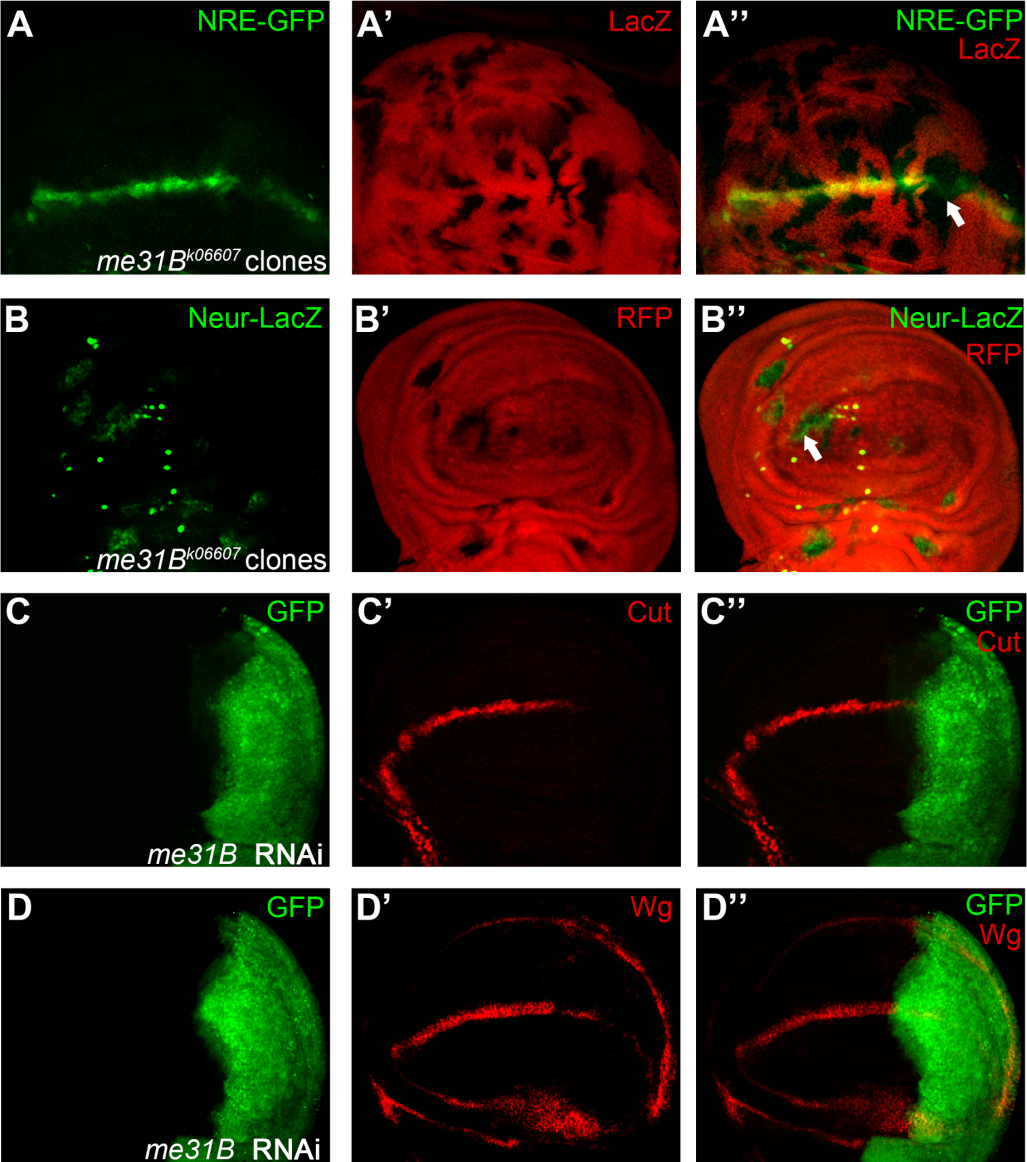
Me31B is required for Notch signaling activation. Expression of Notch signaling reporter NRE-GFP (A) is reduced in *me31B*^*k06607*^ homozygous mutant clones (marked by absence of LacZ staining). In *me31B*^*k06607*^ mutant clones (marked by absence of RFP), Neur-LacZ expressing cells are ectopically induced (B). RNAi knock-down of *me31B* in the posterior compartment (marked by GFP) leads to down-regulation of Cut (C) and Wg (D). Clones presented here are generated in the *Minute* background except for panel B. Representative mutant clones are indicated by arrows.

**Fig 10:**
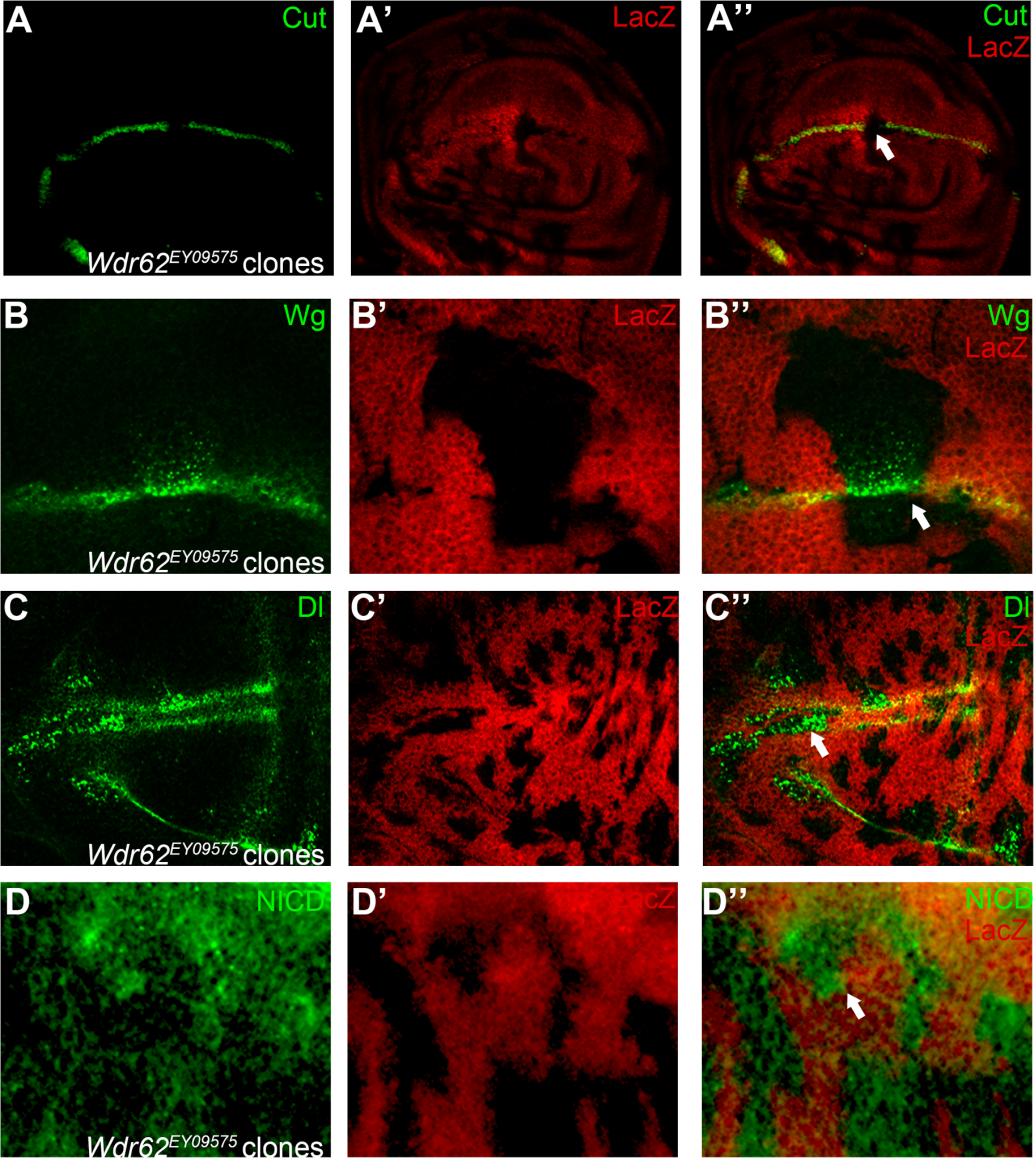
Wdr62 regulates Notch signaling in the wing imaginal disc. In *Wdr62*^*EY09575*^ mutant cells (marked by absence of LacZ staining), expression of Notch signaling target Cut (A) is abolished. Both Wg (B) and Dl (C) protein are accumulated as puncta in *Wdr62*^*EY09575*^ mutant cells. Notch (D) proteins are mildly up-regulated in *Wdr62*^*EY09575*^ homozygous cells. Clones presented here are generated in the *Minute* background. Representative mutant clones are indicated by arrows. Panels B, C and D are higher magnification of a small portion of S6 Fig.

**Fig 11:**
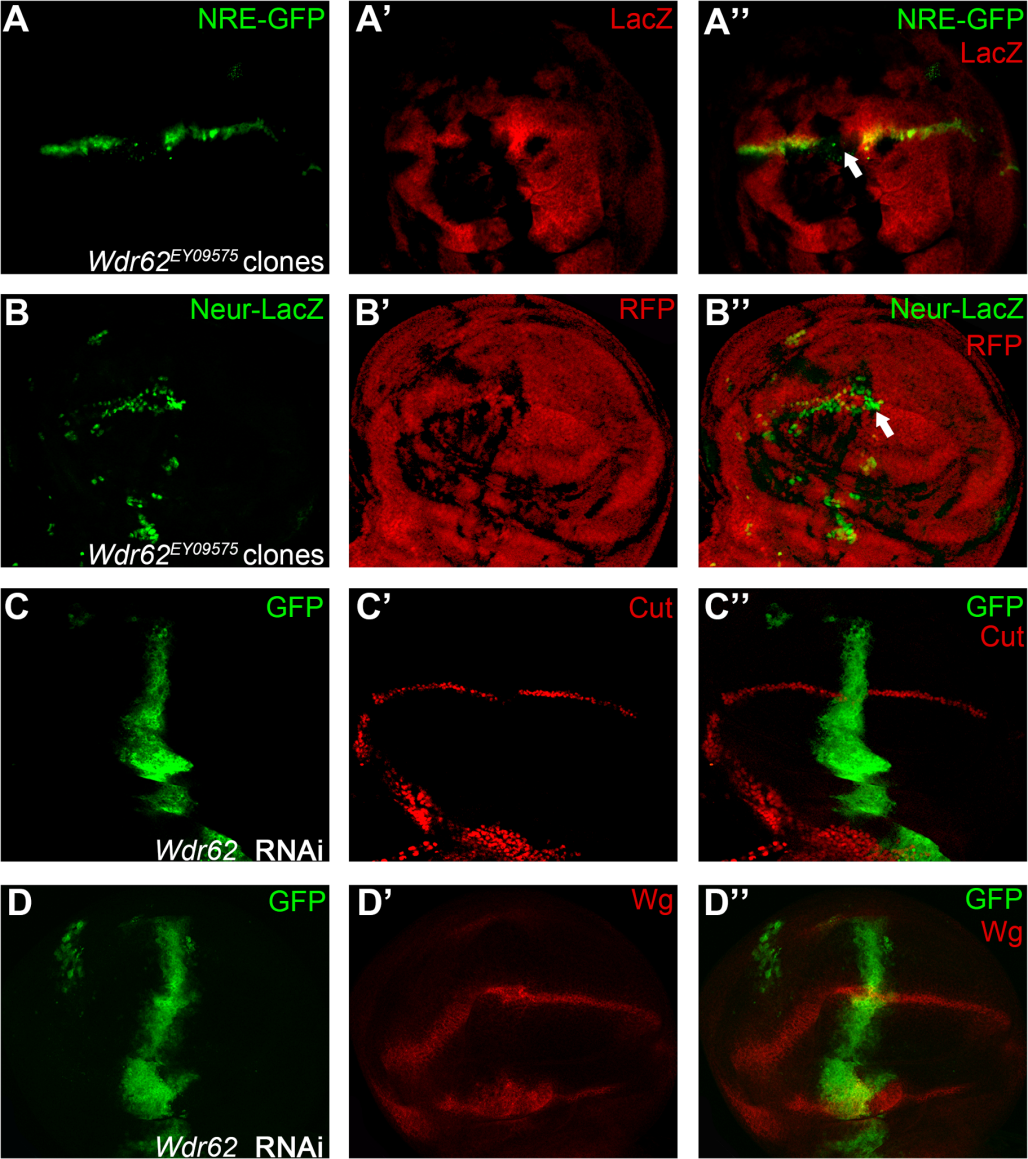
Wdr62 is a positive regulator of Notch signaling activity. Expression of Notch signaling reporter NRE-GFP (A) is reduced in *Wdr62*^*EY09575*^ homozygous mutant cells (marked by absence of LacZ staining). Ectopic induction of Neur-LacZ expressing cells (B) are observed in *Wdr62*^*EY09575*^ mutant clones (marked by absence of RFP). RNAi knock-down of *Wdr62* at the anterior-posterior boundary region (marked by GFP) leads to down-regulation of Cut (C), but not Wg (D). Clones presented here are generated in the *Minute* background except for panel B. Representative mutant clones are indicated by arrows.

Wing discs from third-instar larvae were fixed in 4% paraformaldehyde and labeled with the following primary antibodies: mouse anti-Cut (1:100; 2B10; DSHB), mouse anti-Wingless (1:200; 4D4; DSHB), mouse anti-Notch intracellular domain NICD (1:200; C17.9C6; DSHB), mouse anti-Delta (1:200; C594.9B; DSHB) and rabbit anti-LacZ (1:4000; Cappel). Alexa fluor-conjugated secondary antibodies (1:400; Invitrogen) were used. The fluorescence images were acquired with an Olympus FV1000 or Leica SP8 confocal microscope and processed in Photoshop. Minor adjustments (brightness and/or contrast) were done in Photoshop. Representing single focal plane pictures were shown in Figs 1, 2, 3A–3C, 7A, 9C, 9D, 10A–10C and 11B–11D. Projection of z-stacks were generated by ImageJ and shown in Figs 3D, 4, 5, 6, 7B–7D, 8, 9A, 9B, 10D and 11A.

Adult wings were dissected and mounted as described previously [9]. The images of adult wings were acquired with a Leica DMIL inverted microscope equipped with a QImaging QICAM Fast 1394 digital camera.

## Results and discussion

### Genetic mosaic screen identifies genes modulating Notch signaling during fly wing development

Many Notch signaling modulators have been identified through genome-wide mutagenesis screens, RNAi screens and modifier screens. Therefore, it is unlikely to find new Notch regulators using similar strategies [2–5]. We designed a small scale screen using the Flippase (FLP)/FLP Recognition Target (FRT) genetic mosaic technique [10]. We screened a collection of lethal mutations, based on the knowledge that most Notch regulators are essential for larval development in *Drosophila*. We took advantage of a modified Ubx-promoter driven FLP transgene that expresses the FLP recombinase in the developing wing imaginal disc, and thus mediates mitotic recombination in a labor-free manner [11].

We screened 451 BruinFly lines with lethal mutations in genes located on the left arm of chromosome II [7]. While the initial focus of our screen was to find specific regulators for wing margin formation, we observed that many of these lines led to developmental defects in the wing. In total, 60 BruinFly lines displayed wing phenotypes when homozygous clones were present (S1 Table). The mutants were categorized into six classes based on similarity of resulting phenotypes: nicked wing margin, abnormal vein pattern, tissue necrosis, blistered wing, folded wing and club-like wing (S1 Fig). Mutants causing “nicked wing margin” were further analyzed as this represents the classical phenotype associated with dysregulation of Notch signaling.

Among the 30 mutants that showing notched wing phenotypes, five of them represent mutations of genes that are involved in Decapentaplegic (Dpp) and Epidermal Growth Factor Receptor (EGFR) signaling pathways. During fly wing development, Dpp protein functions as a morphogen that controls organ growth and vein differentiation. The type I receptor Thickveins (Tkv) and downstream transcription factor Mothers against Dpp (Mad) are essential for Dpp signaling transduction [12]. We found that mutations of *Tkv* and *Mad* caused vein thickening as well as margin notches in the wing (S1 Fig). Vein thickening is a stereotypical developmental defect resulted from reduction of Dpp signaling [12]. And the notched wing phenotypes might reflect the ability of Dpp signaling to regulate Wingless (Wg) signaling activity during wing margin formation [13, 14]. *Star* (*S*) encodes a transmembrane protein that facilitates processing and secretion of EGFR ligands [15]. In wings bearing *S* mutant clones, we observed missing of veins, and occasionally wing margin notches (S1 Fig). The vein missing phenotype resembles *S* loss-of-function alleles [16]. Again, EGFR pathway interacts with Wg signaling to regulate wing margin formation [17, 18]. Thus, these lines were not scored as specific Notch regulators.

Directly controlled by Notch signaling activity, both Cut and Wg are expressed in cells located at the dorsal-ventral (DV) boundary of the wing disc (S2 Fig). Therefore, expression of Cut and Wg were examined in the mitotic clones for the rest 25 mutant alleles. We found that 5 of these mutants gave rise to very tiny clones which are much smaller than their twin-spot sister clones, suggesting that these genes are crucial for cell viability during wing development (S2 Table). Another 9 mutants resulted in no obvious changes in the expression of neither Cut nor Wg (S2 Table). These genes likely regulate wing margin formation through other signaling pathways or cellular events [6, 19].

At last, we isolated 9 genes from 11 mutant lines that were qualified as Notch signaling regulators during fly wing development (Fig 1). Given the variety of genes uncovered in our screen, we categorized them into three groups based on previous studies and importance (Table 1). Three of these genes are well-known regulators of the Notch signaling. Four of them were related with Notch signaling in other tissues, but their roles for regulation of Notch activity during wing development were poorly studied. More importantly, we identified two novel modulators of the Notch signaling pathway.

**Table 1:** Summary of Notch signaling regulators revealed by screen.

Gene Symbol	BruinFly Allele	Wing*	Other tissues*	Group
*Su(H)*	*Su(H)*^*k07904*^	Yes [1, 5]	Yes [1, 5]	I
*Hrb27C*	*Hrb27C*^*EY12571*^ *Hrb27C*^*k02814*^ *Hrb27C*^*f04375*^	Yes [23–25]	Yes [24]	I
*eIF3h*	*eIF3h*^*K09003*^	Yes [9]	No	I
*Vha68-2*	*Vha68-2*^*s4214*^	No	Yes [28]	II
*VhaSFD*	*VhaSFD*^*EY04644*^	No	Yes [28]	II
*Pp2A-29B*	*Pp2A-29B*^*EP2332*^	No	Yes [39, 40]	II
*mts*	*mts*^*s5286*^	No	Yes [39, 40]	II
*me31B*	*me31B*^*k06607*^	No	No	III
*Wdr62*	*Wdr62*^*EY09575*^	No	No	III

* Previous studies of each gene for their roles in Notch signaling regulation in the wing or other tissues are summarized.

#### Group I: Notch signaling components involved in wing development

Gene encoding Su(H) was identified by the screen, which was expected as Su(H) is a core component required for Notch target gene activation [1, 5]. We observed a very high rate of marginal notches in mosaic wings harboring the P-element insertion allele *Su(H)*^*k07904*^ (Fig 1B). To confirm the effect of *Su(H)*^*k07904*^ in Notch signaling transduction, we generated homozygous *Su(H)*^*k07904*^ mutant clones in the wing discs and analyzed the expression of two Notch target genes, namely Cut and Wg as a readout. The expression of Cut and Wg were completely absent in cells homozygous for *Su(H)*^*k07904*^, which are in agreement with previous findings that Su(H) facilitates Notch target genes transcription (S3 Fig). We found that Notch itself was not regulated by Su(H) (S3 Fig). Interestingly, Dl expression was modestly reduced in *Su(H)*^*k07904*^ clones located at the DV boundary (S3 Fig). Our results suggest that Su(H) may play additional role in Dl expression regulation during fly wing development.

We also found wing margin nicking phenotypes in three BruinFly alleles corresponding to *Hrb27C/Hrp48* (Fig 1C–1E). The *Hrb27C*^*EY12571*^ allele was chosen for further characterization because it gave the highest percentage of wing notch phenotypes among three alleles. Reductions of Notch signaling targets were evident in *Hrb27C*^*EY12571*^ mutant clones (Fig 2A and 2B). Hrb27C is an abundant, essential RNA binding protein that functions in RNA splicing, localization and translation control [20–22]. During fly wing development, Hrb27C was shown to promote Notch expression [23]. Accordingly, Notch levels were reduced in *Hrb27C*^*EY12571*^ mutant clones (Fig 2C). The exact biochemical mode of action of Hrb27C in Notch signaling regulation is still elusive. It was proposed that Hrb27C regulates Notch expression through the female determinant Sex-lethal (Sxl), but neither the splicing nor stability of *Sxl* mRNAs were modulated by Hrb27C [23, 24]. Recently, Hrb27C was identified as an interacting partner of Deltex and they regulate Notch protein independent of Sxl [25]. Whether other core components of Notch pathway were influenced by Hrb27C was unknown. We found that Dl expression was also slightly reduced in cells homozygous of *Hrb27C*^*EY12571*^ when clones are located at the DV boundary (Fig 2D). Thus, the role of Hrb27C in Notch signaling regulation appears to be more complex than expected and awaits further investigation.

The *Drosophila* eIF-3p40/eIF3h protein is a subunit of the eukaryotic translation initiation factor 3 (eIF3) complex [26]. A regulatory role of eIF3 complex in Notch pathway was discovered in our previous RNAi screen [9]. Here we report that mosaic clones of the BruinFly allele *eIF3h*^*K09003*^ led to marginal defects in the wing (Fig 1F). Consistent with the adult phenotype, Notch signaling activity was diminished in *eIF3h*^*K09003*^ clones as revealed by lack of the expression of Cut and Wg (Fig 3A and 3B). We found that Notch expression was substantially reduced upon loss of eIF3h (Fig 3C). These phenotypes are in agreement with the reported RNAi knock-down results [9]. Cells near the DV boundary express highest levels of Dl due to the positive feedback loop of the Notch signaling [1, 4, 6]. We found that Dl expression were dampened in *eIF3h*^*K09003*^ mutant cells only when clones were located at the DV boundary, likely resulting from reduced Notch signaling activity (Fig 3D). Taken together, our results suggest that eIF3h is a *bona fide* Notch signaling regulator. It is possible that eIF3h is required for translation initiation of Notch protein. Alternatively, eIF3h may possess activities other than translation initiation, as is the case for eIF3f, another subunit of the eIF3 complex which regulates ubiquitination of Notch protein [27].

#### Group II Notch signaling modulators studied in other tissues

Genes in this group encode subunits of two protein complexes with crucial enzyme activities, and their roles in Notch signaling during wing development were not thoroughly studied.

Our screen isolated two vacuolar ATPase (V-ATPase) genes, *Vha68-2* and *VhaSFD* as Notch signaling regulators in wing development. Mutant clones of *Vha68-2*^*s4214*^ and *VhaSFD* ^*EY04644*^ caused notching of the wing margin (Fig 1G and 1H) and thickening of the wing veins (Fig 1H). In agreement with the adult phenotype, we observed loss of Cut and Wg expression in *Vha68-2* ^*s4214*^ and *VhaSFD*^*EY04644*^ mutant clones (Fig 4A and 4B and Fig 5A and 5B). Clones of *Vha68-2* ^*s4214*^ and *VhaSFD*^*EY04644*^ mutant cells also displayed various degrees of Notch and Dl accumulation in intracellular puncta (Fig 4C and 4D, Fig 5C and 5D and S4 Fig). We noticed that the *Vha68-2* and *VhaSFD* mutant phenotypes were also variably penetrant in the eye disc, with some cells showing very mild defects [28]. This could be explained by hypomorphy of the alleles or functional redundancy with other V-ATPase subunits. Despite the phenotypic variability, these results support the view that V-ATPase activity is positively required for Notch signaling. The V-ATPases are conserved multi-subunit ATP-driven proton pumps that present in the endo-membranes of all cells [29]. As demonstrated in *Drosophila*, V-ATPases play a multiplicity of roles during animal development [30–33]. Although some V-ATPase subunits also fulfill specialized roles [34], the similarity of loss-of-function phenotypes suggest that Vha68-2 and VhaSFD likely function in the same complex to regulate Notch signaling in various epithelia tissues. In general, the V-ATPase controls endosomal acidification and is a major regulator of membrane protein localization [28, 31, 35]. Membrane bound signaling molecules such as Dl are transported by the endocytic pathway [36]. Therefore, it is reasonable to predict that localization of Dl is also regulated by Vha68-2 and VhaSFD.

Two subunits of the serine/threonine protein phosphatase 2A (PP2A) holoenzyme were identified in our screen. PP2A consists of a scaffolding A subunit, a regulatory B subunit, and a catalytic C subunit [37]. We found that the A (CG17291/ PP2A-29B) and C (Mts) subunits were required for wing margin formation, mutant clones of either subunit resulted in wing notches (Fig 1I and 1J). As these phenotypes are reminiscent of the loss of Notch function, we examined the activity of Notch signaling in the wing discs. We observed that cells in the *Pp2A-29B*^*EP2332*^ and *mts*^*s5286*^ homozygous mutant clones lack expression of Cut and Wg at the DV boundary (Fig 6A and 6B and Fig 7A and 7B). We observed mild down-regulation of Notch protein in *Pp2A-29B*^*EP2332*^ and *mts*^*s5286*^ mutant cells (Fig 6C and Fig 7C). Dl proteins were maintained at normal levels in *Pp2A* mutant clones (Fig 6D and Fig 7D). It has been shown that removing either the A or the C subunit destabilizes PP2A and reduces its activity [38]. Thus, we conclude that PP2A activity is required for proper Notch signaling output during wing development. The A and C subunits are essential for the activity of PP2A, but the substrate specificity are determined by the variable regulatory B subunits [37]. The fly genome encodes one A, one C, and four B subunits. One of the B subunits, Wdb, was shown to inhibit Notch signaling activity by targeting Enhancer of split M8, an effector of Notch signaling during eye development [39]. Over-expression of Wdb and mts in the developing wing disc led to wing margin loss and ectopic macrochaetes on the notum, also suggesting an inhibitory role for Wdb/PP2A in Notch signaling [39, 40]. However, a recent RNAi screen suggested that PP2A-29B, mts and Wdb act to promote of Notch signaling activity in the wing [41]. Further studies are needed to clarify the exact role of PP2A in wing development. PP2A might display tissue specific roles in Notch signaling. It has been demonstrated that PP2A regulates several substrate proteins through distinct B subunits in the Hh pathway [42, 43] and MAPK pathway [44, 45]. It is highly possible that similar strategy is taken by PP2A to regulate Notch signaling pathway.

#### Group III Novel Notch signaling modulators

The BruinFly allele *me31B*^*k06607*^ disrupts function of a RNA binding protein named as Maternal expression at 31B (Me31B) [46]. Me31B is a putative DEAD-box containing RNA helicase that is involved in transport and translational control of oocyte-localizing maternal RNAs [47, 48] as well as neuronal RNAs [49, 50]. During our screen, we found that *me31B* is required for fly wing development. Wing margin notches were observed in flies containing *me31B*^*k06607*^ homozygous clones (Fig 1K). In the larval wing discs, expression of Notch signaling targets Cut and Wg were abolished in *me31B*^*k06607*^ mutant clones (Fig 8A and 8B). Reduction in the levels of Notch targets could result from the ability of Me31B to regulate upstream signaling molecules such as Notch or Dl. To test this, we examined the effect of *me31B*^*k06607*^ mutation on N and Dl protein levels. We found that upon loss of Me31B, both N and Dl proteins were accumulated in intracellular puncta in a subset of mutant cells (Fig 8C and 8D, S5 Fig). These results suggest that the mislocalized Notch and Dl proteins in *me31B*^*k06607*^ cells were incompetent for signaling activity. It has been demonstrated that cellular localization of Notch and Dl proteins are major determinants for their activity [51]. Accumulation of Notch or Dl proteins in different cellular compartments can lead to gain- or loss-of-function defects in a context-dependent manner [4]. For example, when Notch proteins were trapped in the ER, server Notch signaling loss-of-function phenotypes were observed, despite that Notch protein level is highly elevated [52].

We further validated the requirement of Me31B in Notch signaling activation using two independent reporters. The GFP expression in the *NRE-GFP* transgenic line marks the cells with active Notch signaling along the DV boundary of the larval wing disc [53]. In *me31B*^*k06607*^ mutant cells, the GFP expression was obviously reduced (Fig 9A, c.f. S5 Fig). The *neur-lacZ* enhancer-trap insertion labels the sensory organ precursor (SOP) cells, whose fates are repressed by Notch signaling [54]. We found that the number of *neur-lacZ* positive SOPs in *me31B*^*k06607*^ clones was significantly increased (Fig 9B, c.f. S5 Fig). Interestingly, the lacZ proteins are expressed at lower level and exhibit disrupted nuclear localization in *me31B*^*k06607*^ clones. Me31B is known to regulate mRNA stability and protein translational [48, 49]. Therefore, we speculate that Me31B might be required for efficient translation and nuclear transporting of the engineered LacZ protein. Taken together, we conclude that Notch signaling activity is indeed dampened in *me31B*^*k06607*^ mutant cells.

To further demonstrate that these phenotypes are directly caused by *me31B* malfunction, we knocked-down *me31B* expression using transgenic RNAi line in the wing disc. We found that expression of both Cut and Wg were down-regulated in *me31B* RNAi cells (Fig 9C and 9D). Complementation tests were also performed to rule out potential secondary mutations in the *me31B*^*k06607*^ stock. We linked the lethality associated with *me31B*^*k06607*^ to chromosomal region 31B1 using deficiency stocks bearing deletions of different genomic regions (S3 Table). Finally, we obtained white eyed flies carrying the chromosome produced by precise excision of the transposon and found that they no longer produce the wing margin phenotypes. To ensure that the reversion was due to removing of the P-element but not FRT40A itself, we examined the ability of excision stocks to generate mosaic clones in the wing discs. We found that large clones were formed with unaltered expression of Cut at the DV boundary (S5 Fig). Collectively, we provided evidence for a direct relationship between defective Notch signaling and *Me31B* mutation.

Me31B has emerged as a central player in translational repression and mRNA decay [48, 49]. Our preliminary results indicate that both *Notch* and *Dl* mRNAs could be directly targeted by Me31B. Alternatively, Me31B might regulate genes of Notch pathway through microRNA-mediated translational repression in wing discs [49, 55]. In the follicle cells, RNAi knock-down of Me31B was reported to cause up-regulation of Cut during mid-oogenesis [56]. Taken together, we believe that Me31B might regulate Notch signaling in various developmental processes and may function in a tissue specific fashion. Genetic and molecular studies are underway to determine the underlying mechanism of Me31B in Notch signaling regulation.

The second novel Notch signaling modulator gene identified in our screen is *wd40-repeat protein 62* (*Wdr62*). We observed marginal defects in mosaic wings harboring the *Wdr62*^*EY09575*^ mutant allele (Fig 1L). In *Wdr62*^*EY09575*^ mutant cells, expression of Cut was abolished (Fig 10A). The other target of Notch signaling, Wg, responded differently to *Wdr62* mutation. We found that Wg protein accumulated as puncta inside *Wdr62* mutant cells (Fig 10B). The Wg containing pucnta were majorly observed at the apical focal plane of wing disc cells (S6 Fig). Our observations suggest that Wdr62 might be involved in regulation of differential Notch targets expression at the wing margin [57]. Aberrant accumulation of Dl proteins were evident in *Wdr62*^*EY09575*^ mutant cells (Fig 10C), and the effect was not limited to the apical side of wing disc cells as shown by projection of z-stacks (S6 Fig). We also found that Notch expression were mildly up-regulated in *Wdr62*^*EY09575*^ mutant cells (Fig 10D and S7 Fig). In *Wdr62*^*EY09575*^ mutant cells, the *NRE-GFP* expression was diminished (Fig 11A), while the *neur-lacZ* expression was expanded (Fig 11B). Consistently, expression of Cut, but not Wg, was inhibited when *Wdr62* expression was knocked-down by RNAi (Fig 11C and 11D). Using complementation tests, we mapped the lethality associated with *Wdr62*^*EY09575*^ to chromosomal region 22B, matching to the genomic loci of *Wdr62* (S3 Table). Finally, we found that the wing margin and Cut expression defects were reverted by precise excision of the transposon from *Wdr62*^*EY09575*^ (S7 Fig). Taken together, our results suggest that Wdr62 is involved in Notch signaling regulation during fly wing development.

The *Wdr62* gene was identified, relatively recently, as the second most commonly mutated gene in primary microcephaly patients [58–60]. It has been revealed that Wdr62 functions in the regulation of spindle organization, mitotic progression and the duplication and biased inheritance of centrosomes during neural system development [61]. The *Drosophila* ortholog of Wdr62 was essential for larval brain growth, microcephaly defects similar to human patients were observed in *Wdr62* mutants [62, 63]. Notch signaling also plays prominent roles during neural development [64]. Therefore, it is tempting to presume that Wdr62 interacts with Notch signaling to regulate the development of neural as well as somatic tissues. In particular, a functional link between Wdr62 and Notch signaling might be established through asymmetrical segregation of centrosomes. Wdr62 is required to maintain centrosome asymmetry in both *Drosophila* and vertebrate neural stem cells [62, 65, 66]. At the same time, several Notch pathway regulators were found to interact with centrosomes and distribute asymmetrically after mitotic division of neuron progenitor cells [67, 68]. Further studies are needed to clarify whether Wdr62 is capable of regulating Notch signaling during asymmetry cell divisions.

### Conclusions and potential for future studies

The first *Notch* mutant was isolated by T.H. Morgan at 1917, and the *Notch* gene was named after the wing margin loss phenotype [69]. Over the past century, phenotypic studies combined with subsequent genetic and molecular analysis of wing development have been extensively used to identify components of Notch signaling pathway [1–5]. We are encouraged by the fact that new regulators of Notch signaling are discovered by the genetic mosaic system in the wing. Future studies aimed at more deeply characterizing the molecular function of each of the identified targets, particularly the two novel regulators, will broaden our understanding of how Notch signaling is regulated in diverse developmental processes. We believe that somatic mosaic screens will continue to provide valuable insights for understanding Notch signaling regulation [11, 52].

It has been noted that about 5% of the Bruinfly FRT40A stocks might contain second-site mutation alleles of *lethal (2) giant larvae* [*l(2)gl*] [70]. Such mutational genetic background has raised concerns for interpreting genetic studies using these stocks [71]. Importantly, *l(2)gl* is involved in Notch signaling regulation in the developing eye and neuronal cells [72–76]. Therefore, we are obliged to clarify to which extent would the *l(2)gl* mutations confound our screen results. We believe that the presence of *l(2)gl* mutations had little, if any, impact for our screen. This conclusion is based on several important observations. Firstly, *l(2)gl* mutation is unlikely to generate recognizable phenotypes under our experimental conditions. The adult wing blade proper is derived from the pouch region of larval wing imaginal disc. In the pouch area, *l(2)gl* mutant cells growing in mosaic larvae were usually eliminated due to cell death [77, 78]. Therefore, the overall growth and patterning were largely normal in *l(2)gl* mosaic wings [77]. These results suggest that *l(2)gl* mutations would not be recovered in our mosaic screen system. Indeed, we were unable to detect consistent developmental defects for the five Bruinfly FRT40A lines which were shown to contain *lgl* alleles [70]. Secondly, after going through the literatures, we found lack of evidence to support a physiological role of *l(2)gl* in Notch signaling during wing development. When cell competition were alleviated in a Minute surrounding background, *l(2)gl* cells were permissive for survival and clonal growth [78, 79]. Adult wings containing large *l(2)gl* mutant clones displayed a diverse array of developmental defects, but none of them resembled Notch signaling activity disruptions [79]. Similarly, when *l(2)gl* expression was knocked-down by RNAi in the wing disc, the adult tissue displayed significant over-growth but wing margin formation and vein differentiation remained normal [80]. Genome-wide transcriptional profiling found misregulation of several signaling pathways in *l(2)gl* mutant wing disc cells, but failed to detect any significant change in Notch pathway genes [78]. Furthermore, the expression of Notch signaling target gene Wg was unaffected in *l(2)gl* mutant clones generated in the Minute background [78]. Collectively, current findings do not support a direct role of *l(2)gl* in Notch regulation during wing development. It has been shown that Lgl is not involved in regulating Notch signaling in the ovarian follicle cells, strongly suggesting a tissue specific role of Lgl in Notch regulation [81, 82]. Thirdly, among the 11 BruinFly FRT40A lines that identified as Notch regulators in our screen, none of them displayed the neoplastic phenotypes as reported in the *l(2)gl* mutants. Meanwhile, for the two novel regulators identified in our screen, complementation tests and excision experiments established a causal relationship between the mutants and the phenotypes. Taken together, we are confident that the *l(2)gl* mutation background has minimal impact for our study reported here.

## Supporting information

S1 Table Summary of mutant wing phenotypes revealed by screen. (DOCX)

S2 Table Analysis of potential Notch signaling regulators. (DOCX)

S3 Table Complementation analysis of *me31B*^*k06607*^ and *Wdr62*^*EY09575*^ stock. (DOCX)

S1 Fig Various wing phenotypes are discovered in the screen. (TIF)

S2 Fig Notch signaling components display stereotypical distribution patterns. (TIF)

S3 Fig Su(H) is required for Notch signaling activity in the developing wing. (TIF)

S4 Fig V-ATPases regulate cellular localization of Notch and Dl. (TIF)

S5 Fig Me31B regulates cellular localization of Notch and Dl. (TIF)

S6 Fig Wdr62 regulates cellular localization of Wg and Dl. (TIF)

S7 Fig *Wdr62*^*EY09575*^ precise excision lines are restored to wild-type. (TIF)
