# Supplementary material for: A genetic mosaic screen identifies genes modulating Notch signaling in Drosophila
Source: PLoS One. 2018 Sep 20;13(9):e0203781. doi: 10.1371/journal.pone.0203781 (PMC6147428; doi:10.1371/journal.pone.0203781)
Supplement: S1 Table — (DOCX) [file pone.0203781.s001.docx]

**Table S1 Summary of mutant wing phenotypes revealed by screen**

| **DGRC Stock Number** | **Gene Symbol** | **Gene Name** | **BruinFly Allele** |
| --- | --- | --- | --- |
| **Nicked Wing Margin** |  |  |  |
| 111151 | *rgr* | *regular* | *rgr^k02605^* |
| 114524 | *MED20* | *Mediator complex subunit 20* | *MED20^f00955^* |
| 111435 | *mRpL4* | *mitochondrial ribosomal protein L4* | *mRpL4^k14608^* |
| 114528 | *CG7806* | *CG7806* | *CG7806^f02044^* |
| 114545 | *Gas41* | *Gas41* | *Gas41^f05565^* |
| 111285 | *kis* | *kismet* | *kis^k10237^* |
| 111304 | *kis* | *kismet* | *kis^k11324^* |
| 111485 | *kis* | *kismet* | *kis^BG01657^* |
| 114432 | *l(2)gd1* | *lethal (2) giant discs 1* | *l(2)gd1^EY04750^* |
| 111584 | *nub* | *nubbin* | *nub^KG07049^* |
| 114578 | *kuz* | *kuzbanian* | *kuz^EY03488^* |
| 111311 | *RFeSP* | *Rieske iron-sulfur protein* | *RFeSP^k11704^* |
| 114537 | *PrBP* | *Prenyl-binding protein* | *PrBP^f04175^* |
| 114553 | *Caper* | *Caper* | *Caper^f07714^* |
| 111141* | *tkv* | *thickveins* | *tkv^k01302^* |
| 111360* | *tkv* | *thickveins* | *tkv^k16713^* |
| 111123* | *Mad* | *Mothers against dpp* | *Mad^k00237^* |
| 114393* | *Mad* | *Mothers against dpp* | *Mad^kG00581^* |
| 114586* | *S^EY11186^* | *Star* | *Star^EY11186^* |
| 111227 | *Su(H)* | *Suppressor of Hairless* | *Su(H)^k07904^* |
| 111072 | *Hrb27C* | *Heterogeneous nuclear ribonucleoprotein at 27C* | *Hrb27C^k02814^* |
| 114656 | *Hrb27C* | *Heterogeneous nuclear ribonucleoprotein at 27C* | *Hrb27C^f04375^* |
| 114679 | *Hrb27C* | *Heterogeneous nuclear ribonucleoprotein at 27C* | *Hrb27C^EY12571^* |
| 111254 | *eIF3h* | *eukaryotic translation initiation factor 3 subunit h* | *eIF-3h^k09003^* |
| 111113 | *Vha68-2* | *Vacuolar H+ ATPase 68 kDa subunit 2* | *Vha68-2^s4214^* |
| 111707 | *VhaSFD* | *Vacuolar H+-ATPase SFD subunit* | *VhaSFD^EY04644^* |
| 114447 | *Pp2A-29B* | *Protein phosphatase 2A at 29B* | *Pp2A-29B^EP2332^* |
| 111114 | *mts* | *microtubule star* | *mts^s5286^* |
| 111201 | *me31B* | *maternal expression at 31B* | *me31B^k06607^* |
| 114478 | *Wdr62* | *WD repeat domain 62* | *Wdr62^EY09575^* |
|  |  |  |  |
| **Abnormal Vein Pattern** |  |  |  |
| 114531 | *Cap-D3* | *Chromosome associated protein D3* | *Cap-D3^f02191^* |
| 111165 | *ThrRS* | *Threonyl-tRNA synthetase* | *Aats-thr^k04203^* |
| 111068 | *capt* | *capulet* | *capt^k01217^* |
| 114519 | *CG31908* | *CG31908* | *CG31908^f00433^* |
| 111234 | *unknown* | *unknown* | *E7-2-17* |
| 111159 | *Sec61α* | *Sec61 α subunit* | *Sec61α^k03201^* |
|  |  |  |  |
|  |  |  |  |
| **Tissue Necrosis** |  |  |  |
| 111290 | *Mhc* | *Myosin heavy chain* | *Mhc^k10423^* |
| 111169 | *milt* | *milton* | *milt^k04704^* |
| 114566 | *CG9253* | *CG9253* | *CG9253^EY02880^* |
|  |  |  |  |
| **Blistered Wing** |  |  |  |
| 111122 | *vkg* | *viking* | *vkg^k00236^* |
| 111247 | *THG* | *tRNA-histidine guanylyltransferase* | *l(2)35Bc^k08808^* |
| 111213 | *TM9SF4* | *Transmembrane 9 superfamily protein member 4* | *TM9SF4^k07245^* |
|  |  |  |  |
| **Folded Wing** |  |  |  |
| 111324 | *l(2)k13506* | *lethal (2) k13506* | *l(2)k13506^k13506^* |
| 111334 | *Cyt-c-p* | *Cytochrome c proximal* | *l(2)k13905^k13905^* |
| 111187 | *vri* | *vrille* | *vri^k05901^* |
| 111203 | *Aac11* | *Aac11* | *Aac11^k06710^* |
| 111206 | *l(2)k07005* | *lethal (2) k07005* | *l(2)k07005^k07005^* |
| 114521 | *CG9249* | *CG9249* | *CG9249^f00835^* |
| 114528 | *CG7806* | *CG7806* | *CG7806^f02044^* |
| 111265 | *l(3)B3-3-21* | *lethal (3) B3-3-21* | *l(3)B3-3-21^1^* |
| 111278 | *ND-PDSW* | *NADH dehydrogenase (ubiquinone) PDSW subunit* | *Pdsw^k10101^* |
| 114540 | *net* | *net* | *net^f04249^* |
| 114558 | *CG10333* | *CG10333* | *CG10333^d02040^* |
| 114584 | *bur* | *burgundy* | *bur^EY11080^* |
| 114591 | *PIG-U* | *Phosphatidylinositol glycan anchor biosynthesis class U* | *PIG-U^DG14109^* |
| 114592 | *TfIIB* | *Transcription factor IIB* | *TfIIB^DG14311^* |
| 111145 | *Ddc* | *Dopa decarboxylase* | *Ddc^k02104^* |
| 111064 | *esg* | *escargot* | *esg^k00606^* |
|  |  |  |  |
| **Club-like Wing** |  |  |  |
| 111118 | *Btk29A* | *Btk family kinase at 29A* | *Btk29A^k00206^* |
| 114462 | *Btk29A* | *Btk family kinase at 29A* | *Btk29A^EP2167^* |

* These lines display both “nicked wing margin” and “abnormal vein pattern” phenotypes.
