# Supplementary material for: A genetic mosaic screen identifies genes modulating Notch signaling in Drosophila
Source: PLoS One. 2018 Sep 20;13(9):e0203781. doi: 10.1371/journal.pone.0203781 (PMC6147428; doi:10.1371/journal.pone.0203781)
Supplement: S2 Table — (DOCX) [file pone.0203781.s002.docx]

| **DGRC Stock Number** | **BruinFly Allele** | **Phenotypes in Somatic Clones** |
| --- | --- | --- |
| 111151 | *rgr^k02605^* | Clones too small |
| 114524 | *MED20^f00955^* | Clones too small |
| 111435 | *mRpL4^k14608^* | Clones too small |
| 114528 | *CG7806^f02044^* | Clones too small |
| 114545 | *Gas41^f05565^* | Clones too small |
| 111285 | *kis^k10237^* | No defects |
| 111304 | *kis^k11324^* | No defects |
| 111485 | *kis^BG01657^* | No defects |
| 114432 | *l(2)gd1^EY04750^* | No defects |
| 111584 | *nub^KG07049^* | No defects |
| 114578 | *kuz^EY03488^* | No defects |
| 111311 | *RFeSP^k11704^* | No defects |
| 114537 | *PrBP^f04175^* | No defects |
| 114553 | *Caper^f07714^* | No defects |
| 111227 | *Su(H)^k07904^* | Defective Notch signaling activity |
| 111072 | *Hrb27C^k02814^* | Defective Notch signaling activity |
| 114656 | *Hrb27C^f04375^* | Defective Notch signaling activity |
| 114679 | *Hrb27C^EY12571^* | Defective Notch signaling activity |
| 111254 | *eIF-3h^k09003^* | Defective Notch signaling activity |
| 111113 | *Vha68-2^s4214^* | Defective Notch signaling activity |
| 111707 | *VhaSFD^EY04644^* | Defective Notch signaling activity |
| 114447 | *Pp2A-29B^EP2332^* | Defective Notch signaling activity |
| 111114 | *mts^s5286^* | Defective Notch signaling activity |
| 111201 | *me31B^k06607^* | Defective Notch signaling activity |
| 114478 | *Wdr62^EY09575^* | Defective Notch signaling activity |

**Table S2 Analysis of potential Notch signaling regulators**
