# Supplementary material for: A genetic mosaic screen identifies genes modulating Notch signaling in Drosophila
Source: PLoS One. 2018 Sep 20;13(9):e0203781. doi: 10.1371/journal.pone.0203781 (PMC6147428; doi:10.1371/journal.pone.0203781)
Supplement: S3 Table — (DOCX) [file pone.0203781.s003.docx]

**Table S3 Complementation analysis of *me31B^k06607^* and *Wdr62^EY09575^* stock**

| **BruinFly Allele** | **Gene Location** | **Deficiency Stock** | **Deficiency Region** | **Complementation of lethality** |
| --- | --- | --- | --- | --- |
| *me31B^k06607^* | 31B1-31B1 | 9503  BL* | 31B1-31D9 2L:10209408 -10333704 | No |
| *me31B^k06607^* | 2L:10239341-  10242172 | 150096 KY^#^ | 31B1-32A5 2L:10247014 -10732704 | Yes |
| *me31B^k06607^* |  | 7818  BL* | 31A3-31B1  2L:10134181- 10198992 | Yes |
| *Wdr62^EY09575^* | 22B4-22B6 | 7778  BL* | 22B1-22B5  2L:1716977-1909976 | No |
| *Wdr62^EY09575^* | 2L:1884622-  1944289 | 7779  BL* | 22B2-22B8 2L:1737960- 2010136 | No |
| *Wdr62^EY09575^* |  | 8000  BL* | 22B5-22D1 2L:1911627-2175599 | No |
| *Wdr62^EY09575^* |  | 150067  KY ^#^ | 22B8-22D4  2L: 1985930-2,222,065 | Yes |

* These stocks are obtained from Bloomington Stock Center.

^#^ These stocks are obtained from Kyoto Stock Center.
