# Supplementary figures and images for: A genetic mosaic screen identifies genes modulating Notch signaling in Drosophila
Source: PLoS One. 2018 Sep 20;13(9):e0203781. doi: 10.1371/journal.pone.0203781 (PMC6147428; doi:10.1371/journal.pone.0203781)

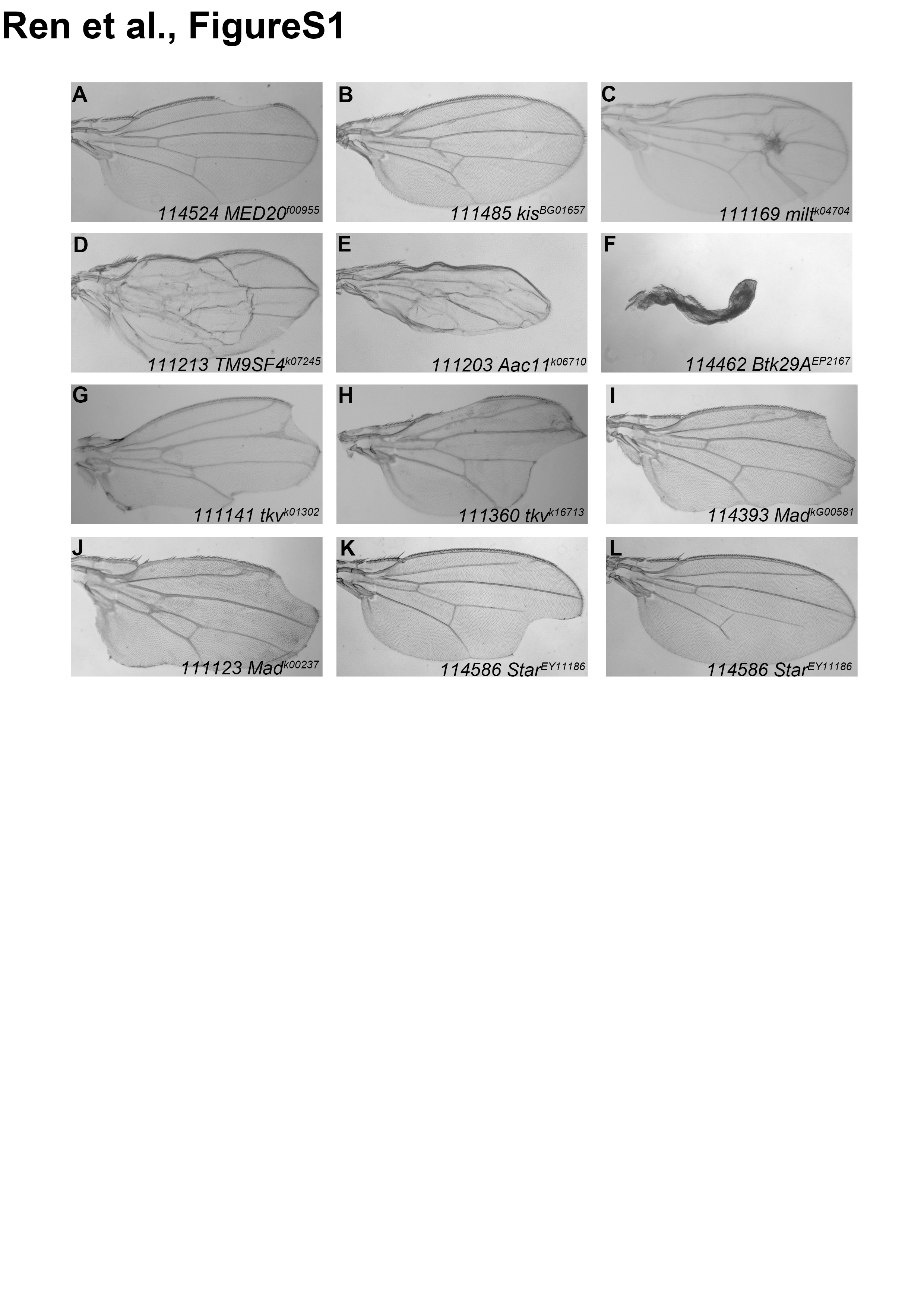

Supplement: S1 Fig — (TIF) [file pone.0203781.s004.tif]

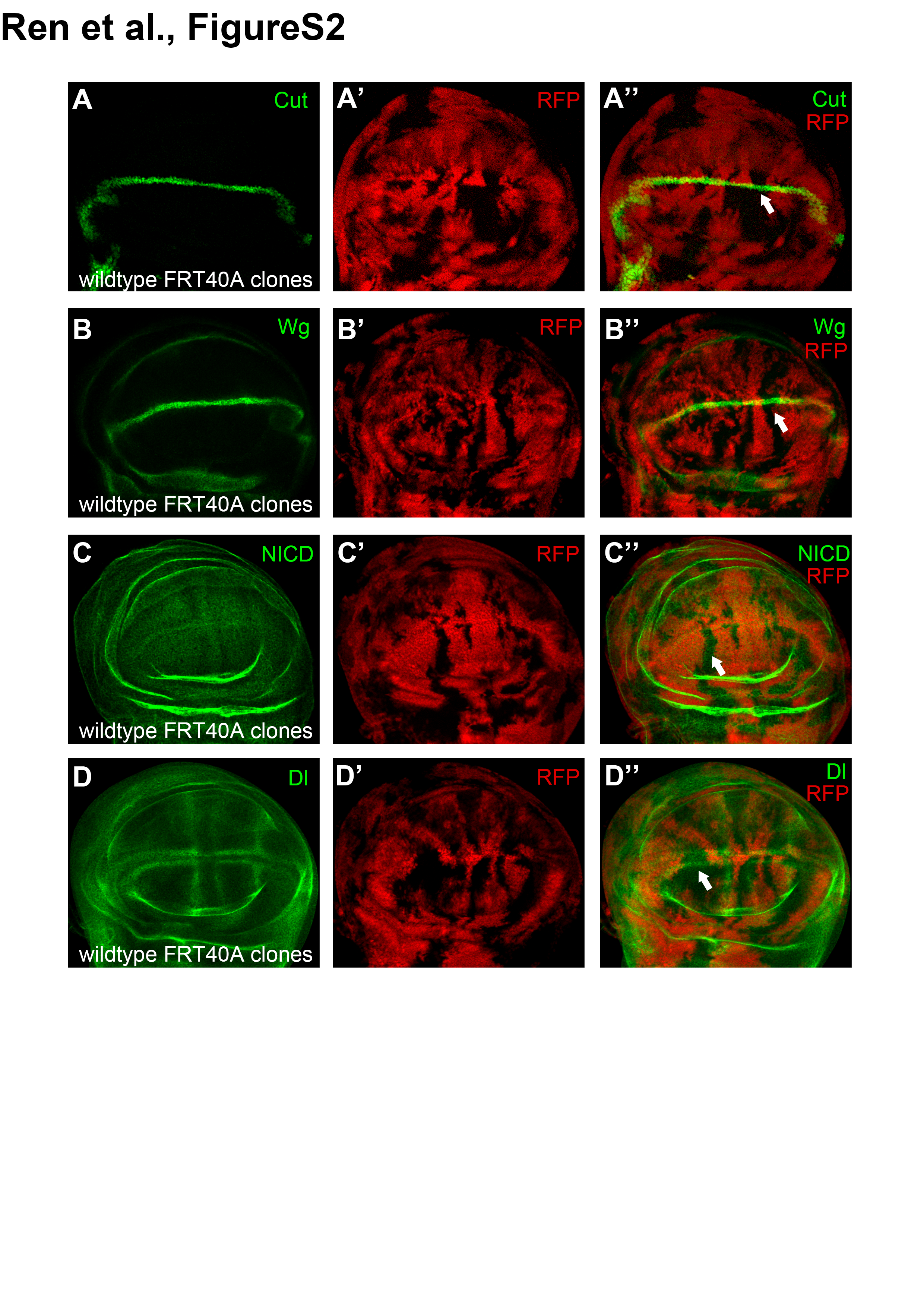

Supplement: S2 Fig — (TIF) [file pone.0203781.s005.tif]

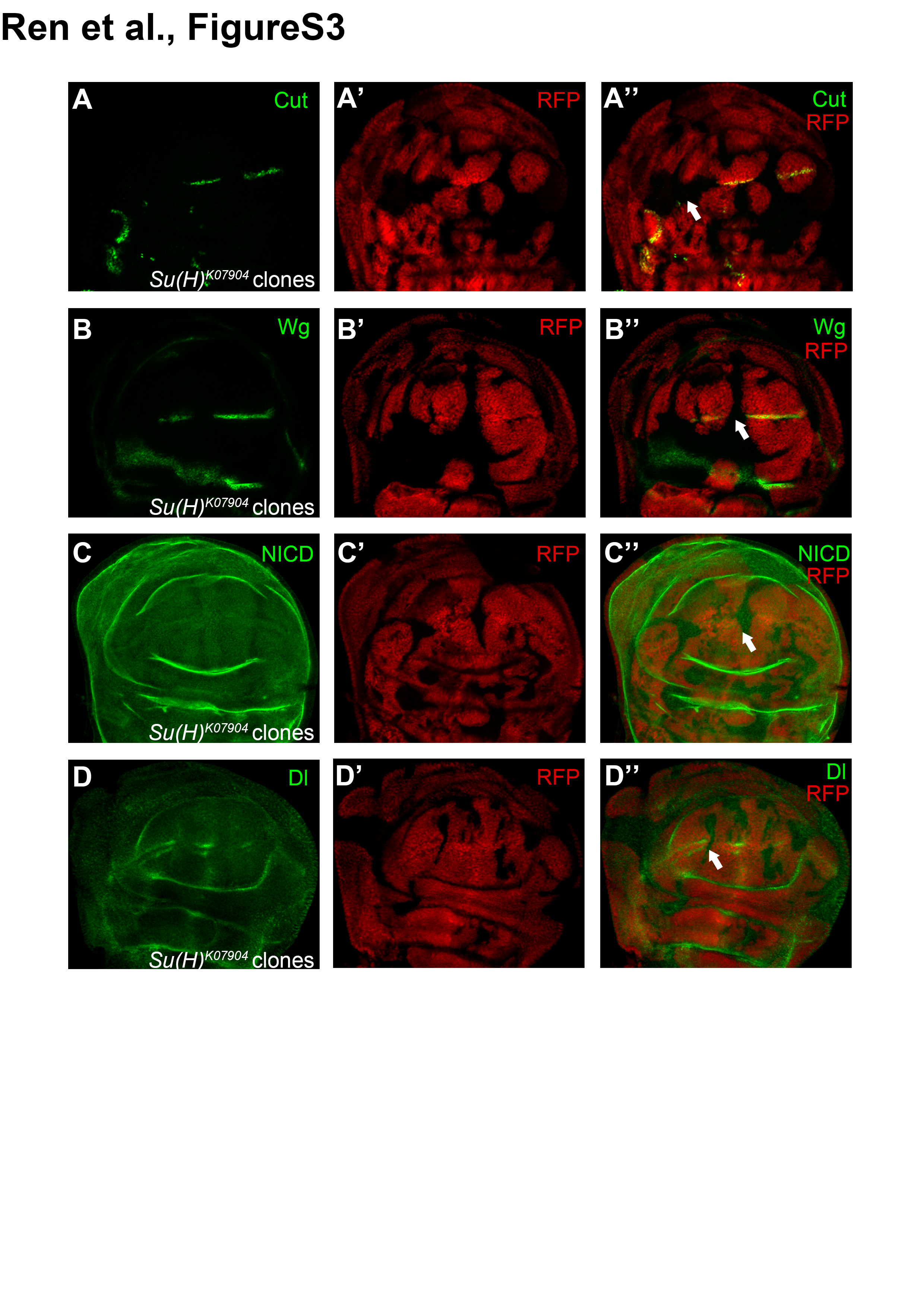

Supplement: S3 Fig — (TIF) [file pone.0203781.s006.tif]

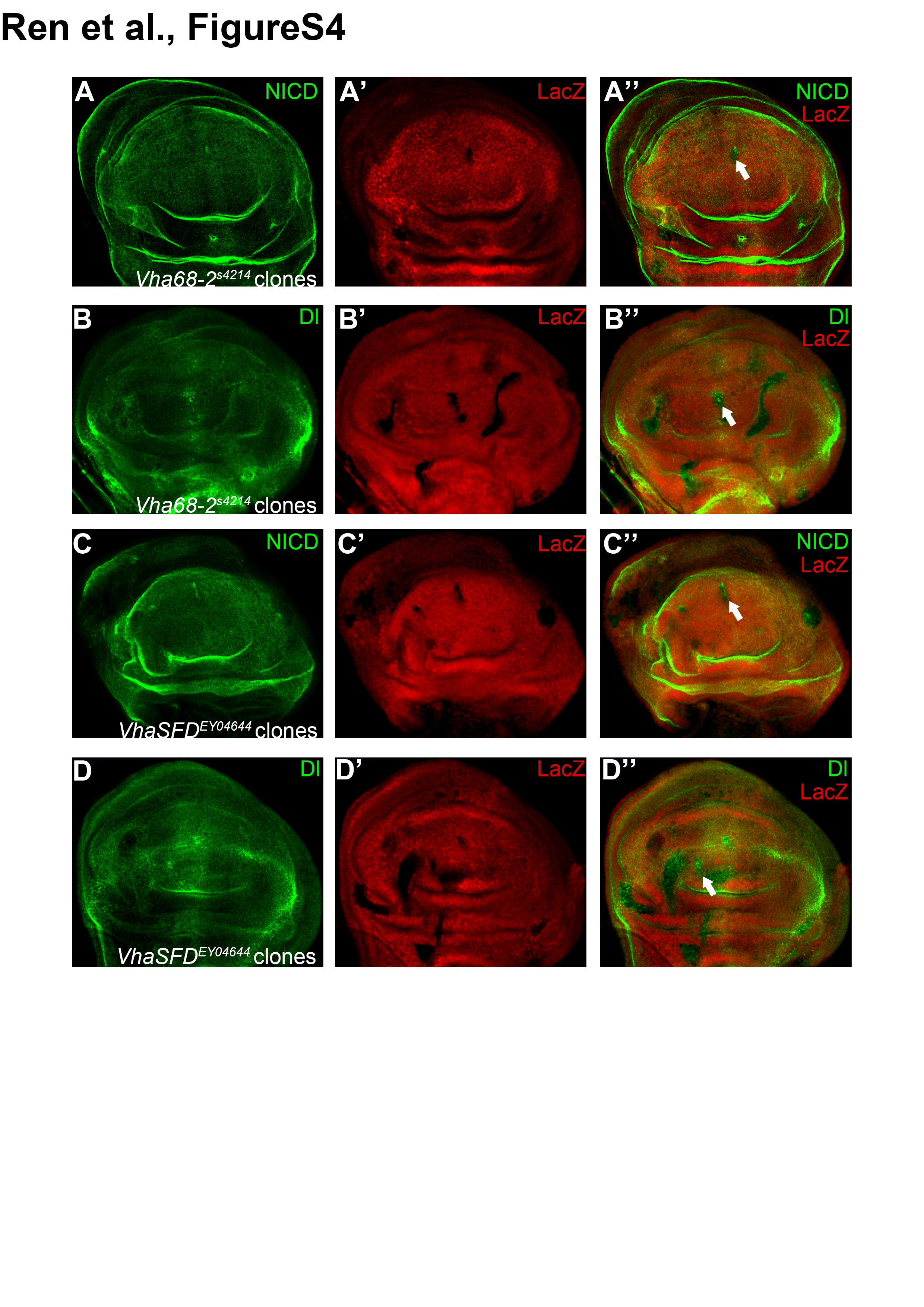

Supplement: S4 Fig — (TIF) [file pone.0203781.s007.tif]

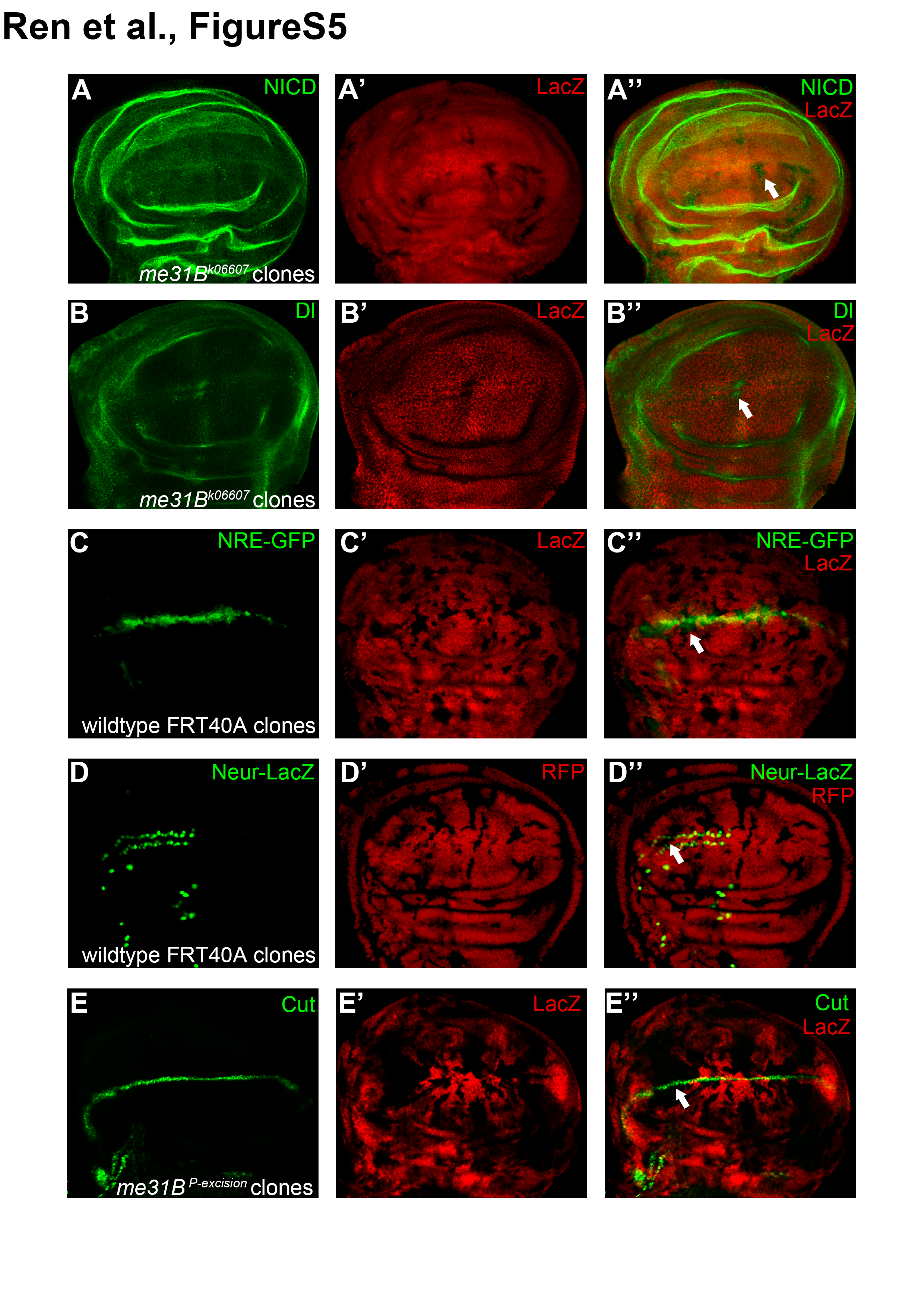

Supplement: S5 Fig — (TIF) [file pone.0203781.s008.tif]

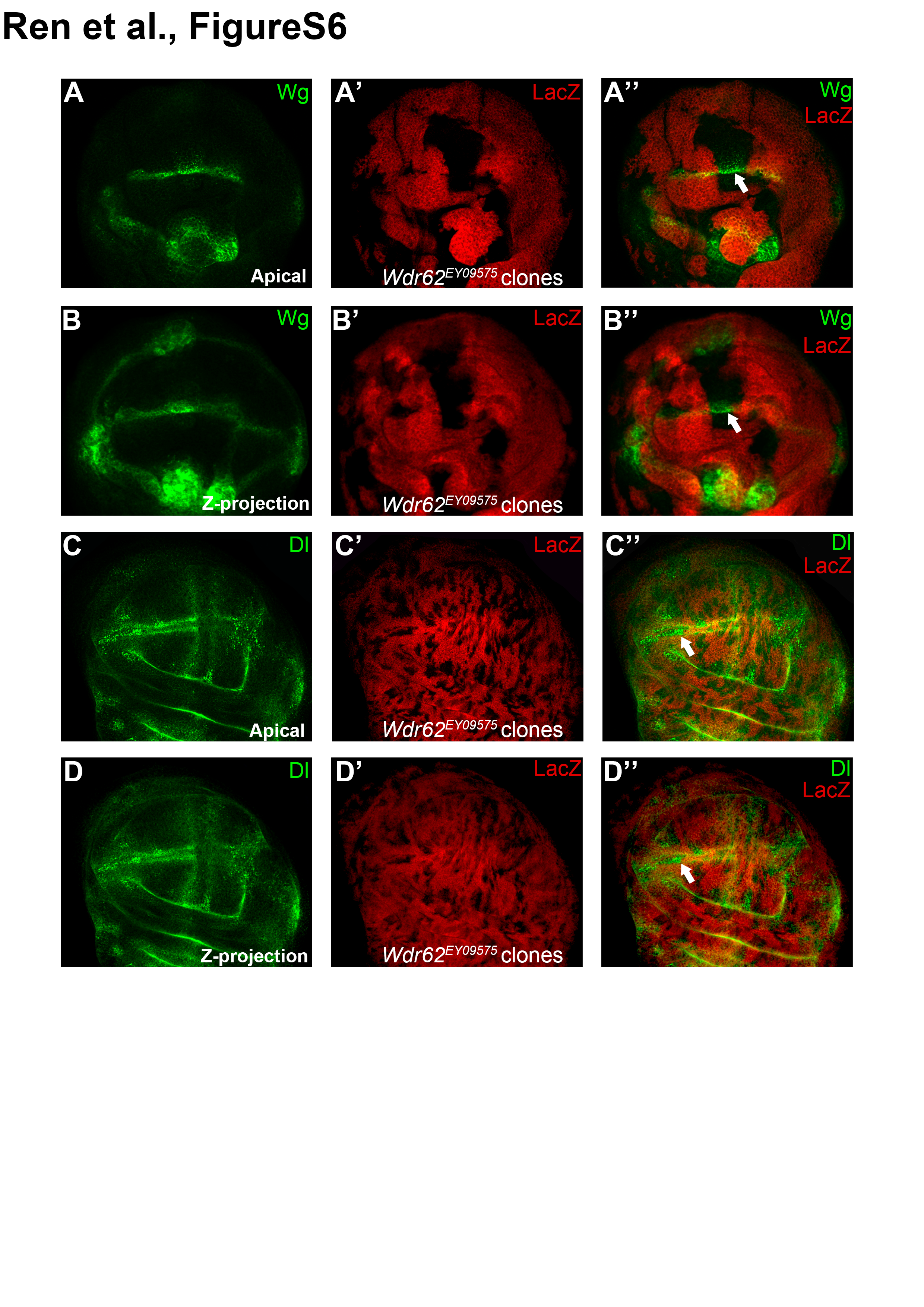

Supplement: S6 Fig — (TIF) [file pone.0203781.s009.tif]

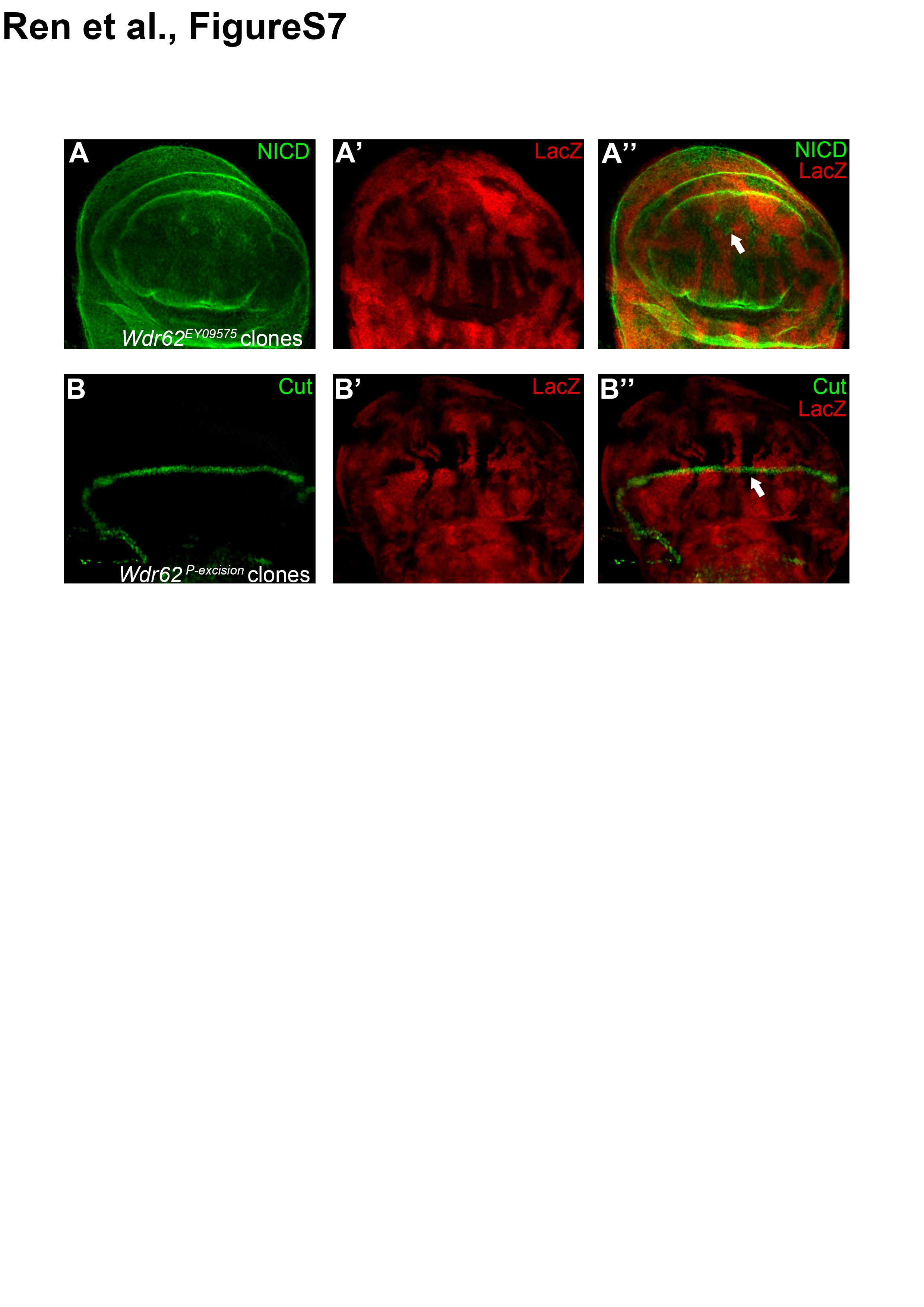

Supplement: S7 Fig — (TIF) [file pone.0203781.s010.tif]
